# Metabolic framework of spontaneous and synthetic sourdough metacommunities to reveal microbial players responsible for resilience and performance

**DOI:** 10.1186/s40168-022-01301-3

**Published:** 2022-09-14

**Authors:** Francesco Maria Calabrese, Hana Ameur, Olga Nikoloudaki, Giuseppe Celano, Mirco Vacca, Wilson JFLemos Junior, Caterina Manzari, Fabienne Vertè, Raffaella Di Cagno, Graziano Pesole, Maria De Angelis, Marco Gobbetti

**Affiliations:** 1 Department of Soil, Plant and Food Science, University of Bari Aldo Moro, Bari, Italy; 2 Faculty of Science and Technology, Libera Università Di Bolzano, Piazza Università 5, 39100 Bolzano, Italy; 3 Department of Biosciences, Biotechnology and Biopharmaceutics, University of Bari Aldo Moro, Bari, Italy; 4 Puratos NV, Industrialaan 25, 1702 Groot-Bijgaarden, Belgium

## Abstract

**Background:**

In nature, microbial communities undergo changes in composition that threaten their resiliency. Here, we interrogated sourdough, a natural cereal-fermenting metacommunity, as a dynamic ecosystem in which players are subjected to continuous environmental and spatiotemporal stimuli.

**Results:**

The inspection of spontaneous sourdough metagenomes and transcriptomes revealed dominant, subdominant and satellite players that are engaged in different functional pathways. The highest microbial richness was associated with the highest number of gene copies per pathway. Based on meta-omics data collected from 8 spontaneous sourdoughs and their identified microbiota, we de novo reconstructed a synthetic microbial community SDG. We also reconstructed SMC-SD43 from scratch using the microbial composition of its spontaneous sourdough equivalent for comparison. The KEGG number of dominant players in the SDG was not affected by depletion of a single player, whereas the subdominant and satellite species fluctuated, revealing unique contributions. Compared to SMC-SD43, SDG exhibited broader transcriptome redundancy. The invariant volatilome profile of SDG after in situ long-term back slopping revealed its stability. In contrast, SMC-SD43 lost many taxon members. Dominant, subdominant and satellite players together ensured gene and transcript redundancy.

**Conclusions:**

Our study demonstrates how, by starting from spontaneous sourdoughs and reconstructing these communities synthetically, it was possible to unravel the metabolic contributions of individual players. For resilience and good performance, the sourdough metacommunity must include dominant, subdominant and satellite players, which together ensure gene and transcript redundancy. Overall, our study changes the paradigm and introduces theoretical foundations for directing food fermentations.

Video Abstract

**Supplementary Information:**

The online version contains supplementary material available at 10.1186/s40168-022-01301-3.

## Background

Microbiomes are vital components of natural ecosystems whose functions are typically performed not by single species but by metacommunities [1]. The structure, function, resilience, and stability of these metacommunities result from a dynamic interplay of natural selection, historical contingency, and chance events in a manner that remains poorly understood. The ability to predict and reconstruct large multispecies communities requires an understanding of how such microbiomes shape, behave and coexist in natural niches to ultimately design de novo functional microbiomes [2–4]. These concepts translate easily to food microbiomes, and specifically to the sourdough microbiome, or “fermentome” (fermenting metacommunity), as we prefer to define this highly variable ecosystem. Here, we experimentally interrogated sourdough as a model dynamic microbial ecosystem. As one of the oldest examples of a natural starter, sourdough is increasingly used for making baked goods, and almost 30 years of research has been carried out to understand and define its performance under abiotic and biotic stresses [5]. The main secret to sourdough performance lies in its microbial diversity. Up to 59 bacterial genera were culturable in sourdoughs, with *Lactobacillus*, under its former taxonomic nomenclature, as the most abundant genus, comprising 82 species, mostly nomadic and heterofermentative [5]. Yeasts are also irreplaceable players in the sourdough metacommunity, with *Saccharomyces cerevisiae* being the most frequently identified [5]. The house microbiota, that of the flours, with resilient and resistant bacteria, also behave as cereal plant endophytes, and those of the other eventual ingredients in the dough serve as the main sources of microbial contamination and spontaneous sourdough fermentation [6]. Frequent back slopping, as a self-renewing community-scale inoculum, and an acidic environment generate a complex microbiome of interacting players (dominants, subdominants and satellites), and the structure and metabolic network of this microbiome are constantly influenced by consistent biotic pressure and spatiotemporal changes. As a result, in most cases, sourdough is prone to instability such that the metabolic networks are constantly threatened. Metacommunity interplay is, therefore, critical in maintaining stability as a precondition for ensuring sourdough functionality. Consequently, sourdough composition and function cannot be estimated or predicted simply by summing its single parts or monitoring the most abundant species [6]. Basic approaches are needed to anticipate the response of the metacommunity, to steer coexisting species to more desirable and resilient states and to design microbiomes *de novo*[1]. The synthetic microbial communities (SMCs) approach constitutes the only route to confirm or invalidate the hypotheses of our study [1, 7]. SMCs allow a more detailed study of the resilience and performance of the metacommunity in simple and well-controlled habitats, where selective and nonselective forces shape the microbiome in mechanistic and quantitative ways. We assume that subdominant players cooperate synergistically with dominant species by expressing accessory genes that are critical for key metabolic pathways and that, although not encoding key functions, satellite members may be metabolically active and equipped with unique genes and enzymes. More generally, gene redundancy should prevent the loss of specific metabolic pathways in the case of microbial perturbance.

**Fig. 1:**
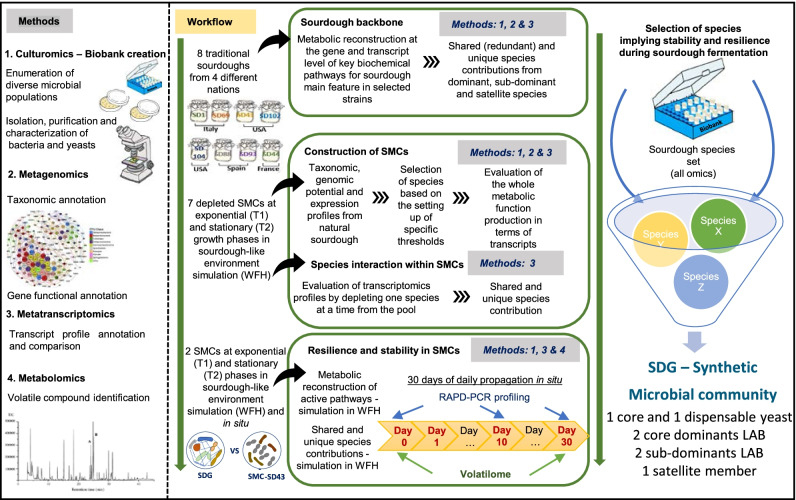
Overview of the multiomics workflow used to decipher the functional redundancy in spontaneous sourdoughs at the metagenomics and metatranscriptomics levels. The inspection of spontaneous sourdough evidenced the metabolic pathways relevant to sourdough biotechnology that guided the phase of de novo reconstruction to obtain a stable and resilient synthetic microbial community

Using a multiomics workflow (Fig. 1), we first deciphered the taxon composition and the functional redundancy of spontaneous sourdoughs at both the metagenomics and metatranscriptomics levels; then, based on these data, we reconstructed the main metabolic pathways that are able to exert key functions within themselves. Subsequently, we used this backbone knowledge to design specifically depleted synthetic microbial communities (SMCs) (where one species at a time was subtracted from the consortium) that guided us in monitoring transcript profile changes during microbial species interactions. As milestones in the workflow, we reconstructed two additional de novo SMCs. The first mimicked a spontaneous sourdough, whereas the second resulted from the funnel selection of species based on omics results. The comparison of their two volatilomes at different back slopping times allowed us to validate the good performance of our selected communities, which could be measured in terms of resiliency, stability, and robustness.

The meta-omics inspection proposed here was useful in laying a theoretical foundation for steering the metacommunity by reconstructing a sourdough fermentome that is as resilient as possible to in situ perturbations.

## Methods

### Traditional and spontaneous sourdoughs

Traditional and spontaneous sourdoughs were obtained from the International Sourdough Library (Puratos, St. Vith, Belgium, https://www.puratos.com/commitments/next-generation/product-heritage/sourdough-library), the only one recognized worldwide. In particular, the 8 sourdoughs used were from Italy (SD1 and SD69), the USA (SD43, SD102 and SD104), Spain (SD88 and SD93) and France (SD44). SD69, SD93 and SD102 were produced with soft wheat flour, with final dough yields (DY) of 143, 160 and 200, respectively. SD43 (DY = 150) and SD104 (DY = 200) were produced with strong soft wheat flour; SD1 (DY = 209), with durum wheat flour; SD44 (DY = 200), with type 80 (specific residual of ash content) soft wheat flour with added honey; and SD88 (DY = 200), with type 80 soft wheat flour with added beer. All sourdoughs were considered mature (constant acidifying capacity) after two back slopping steps, in which the first step included 40% (w/w) inoculum, while the second step used a percentage unique to each sourdough, ranging from 8 to 50%. The back slopping temperature was also unique to each sourdough (from 8 °C up to 28 °C), and the fermentation time ranged from 2 to 24 h. These sourdoughs were the most representative of the International Sourdough Library because of their broad use for making leavened baked goods, and the different geographic provenances reflect countries with a long history and tradition of using sourdough. Supplementary Table S1, Additional File 1 summarizes their origin, ingredients, and technology parameters.

### Culturomics

The eight mature spontaneous sourdoughs were subjected to analyses in triplicate using M17 (30 °C, 48 h), mostly for coccus-shaped lactic acid bacteria, and SDB, mMRS and MRS5 (30 °C, 48 h), mostly for dominant lactobacilli and *Weissella*. Slanetz & Bartley agar (spread plate method, 37 °C, 24–48 h) was used for enterococci, Baird-Parker agar (spread plate method, 30 °C, 48 h) for staphylococci and micrococci, VRBGA for total coliforms, and WA and SDA (30 °C, 48 h) for yeasts. For all media, colonies of bacteria and yeasts with different morphologies were picked and isolated from the penultimate dilution, with the exception of the subdominant culturable lactobacilli, which was isolated from 140 mm diameter plates and considering several decimal dilutions. Isolated colonies were cultivated in broth media and then restreaked onto agar medium (MRS for all lactic acid bacteria and SDA for yeasts) until they were purified. Bacterial and yeast isolates were identified by partial sequencing of the 16S rRNA and 26S rRNA genes, respectively [8, 9]. The identified strains comprising the sourdough biobank were further used upon selection to reconstruct the de novo synthetic microbial communities.

### Shotgun metagenomics and metatranscriptomics sequencing

The DNeasy PowerFood Microbial Kit (Qiagen, Hilden, Germany) was used for DNA extraction [10]. To improve the DNA yield, 100 μl lysozyme (10 mg/ml), 10 μl mutanolysin (20 U/μl [11] and 2 μl Zymolyase (5 U/μl) [12] were added. All library preparations, NGS and quality control steps were performed through RTL-Genomics (Lubbock, Texas). Sequencing was performed on the Illumina MiSeq platform (Illumina Inc., San Diego, CA) applying a depth of coverage of 70X and a fragment size of 300X2 bp paired end (PE) reads. The sequence quality was assessed by inspecting raw read data with FastQC software (https://www.bioinformatics.babraham.ac.uk/projects/fastqc/). Starting from the fastq raw sequencing files, within the SqueezeMeta automated bioinformatics pipeline [13], we used Trimmomatic [14] for adapter removal, trimming and filtering the reads by quality. The details of the bioinformatics workflow are provided below in this section.

Total RNA was extracted using the RNeasy PowerMicrobiome Kit (Qiagen, Hilden, Germany). For each RNA sample, a directional library was prepared using the TruSeq Stranded Total RNA Sample Prep Kit with Ribo-Zero Plus rRNA Depletion Technology (Illumina, San Diego, CA, USA). In detail, RNA-Seq libraries were prepared from 100 ng of total RNA following the manufacturer’s instructions. The cDNA libraries obtained were validated using the High Sensitivity DNA assay (Agilent Technologies, Santa Clara, CA, USA) on a Bioanalyzer 2100 instrument (Agilent Technologies, Santa Clara, CA, USA) and quantified by fluorimetry with the Quant-iTTM PicoGreen® dsDNA Assay Kit (Invitrogen, Carlsbad, CA, USA) on a NanoDrop™ 3300 fluorospectrometer (Thermo Scientific, Waltham, MA, USA). Finally, RNA-Seq libraries were pooled in equimolar ratios and sequenced at a concentration of 1.3 pM on the Illumina NextSeq 500 platform (Illumina, San Diego, CA, USA), generating ca. 20–25 M paired 75 bp reads for each sample.

### SqueezeMeta bioinformatics parameters and nested software

The sequencing data of the 8 sourdough metagenomes/metatranscriptomes were analyzed in silico by using the SqueezeMeta pipeline Version 1.0, July 2019 (https://github.com/jtamames/SqueezeMeta) plus other related ad hoc custom utilities developed to handle the assembly of metagenomes and to obtain genome bins. A step-by-step bioinformatics pipeline comprising software for assembly, annotation and bin statistics was used for sourdough gene and transcript annotation and quantification.

The inspection of annotation data was fundamental for the retrieval of unique/shared functions up to the species taxonomic level. As a dedicated option, we chose to run the metagenome and metatranscriptome assembly using the “coassembly” mode. This procedure allowed us to pool reads from all samples, thus performing a single assembly. Therefore, the derived per sample gene abundances come from mapping the singular read sample set against the coassembly. The assembly was performed using Megahit (v1.1.2) [15], which makes use of succinct de Bruijn graphs [16] to take full advantage of multiple k-mer sizes, optimizing both sensitivity and accuracy. Contig statistics were determined using prinseq [17], which performs rapid quality control and data preprocessing of genomic and metagenomic datasets. The locations of the ribosomal RNA genes in genomes were predicted using Barrnap (BAsic Rapid Ribosomal RNA Predictor) [18], which supports bacteria (5S, 23S, and 16S), archaea (5S, 5.8S, 23S, and 16S) and eukaryotes (5S, 5.8S, 28S, and 18S). The 16S rRNA sequences were taxonomically classified using the RDP classifier [19]. tRNA/tmRNA sequences were predicted using Aragorn [20]. ORFs were predicted using Prodigal [21]. Similarity searches for GenBank [22], eggNOG [23], and KEGG [24] were performed using Diamond [25]. HMM homology searches were performed by HMMER3 [26] for the Pfam database [27]. Read mapping against contigs was performed using Bowtie2 [28], and binning was performed using MaxBin2 [29] and Metabat2 [30]. The combination of binning results was performed using DAS Tool [31]. Bin statistics were computed using CheckM [32]. Pathway prediction for the KEGG [24] and MetaCyc databases was performed using MinPath [33].

### Construction of metagenomics and metatranscriptomics project databases

SqueezeMeta outputs were used to build customized databases viewable in the R environment. Specifically, a set of integrated functions implemented within the SQMtools R package Version 0.3.3 (https://github.com/jtamames/SqueezeMeta/wiki/Using-R-to-analyze-your-SQM-results) allowed us to query specific KEGG output results. The whole metagenomics and metatranscriptomics projects were used to create the two relative databases and then inspected for gene and transcript content and abundances, respectively.

### Synthetic microbial community (SMC) study design

A meta-omics approach was used to design SMCs from the 8 spontaneous sourdoughs. Quantitative data (cell densities) from culturomics were confirmed by the genomic potential and expression levels derived from the metagenomics and metatranscriptomics analyses. By inspecting and merging all these omics data, the sourdough members were classified as core dominant, subdominant (core or dispensable) or satellite species. Starting from this classification, we aimed to de novo reconstruct a potentially stable and resilient SMC (Sourdough Global, SDG). Here, dominant and subdominant species were those harboring at least 20 key genes belonging to carbohydrate/pyruvate and nitrogen metabolism and, at the same time, were shared by at least 50% (4 out of 8) of traditional sourdoughs. Thirteen species out of 49 dominant and subdominant lactic acid bacteria and yeasts met these criteria. Upon considering this filter, further considerations dealing with the overall metabolic capabilities, and worldwide frequency of isolation, SDG comprised 1 core (*Sac. cerevisiae*) and 1 dispensable dominant yeast (*Pichia kudriavzevii*), 2 core dominant (*Lactiplantibacillus plantarum* and *Limosilactobacillus fermentum*) and 2 core subdominant (*Furfurilactobacillus rossiae* and *Pediococcus pentosaceus*) lactic acid bacteria, and 1 satellite species (*Staphylococcus epidermidis*) (Table 1). The species selected to reconstruct the SDG originated from the sourdough biobank and more specifically from SD104, which was the traditional spontaneous sourdough with the highest species biodiversity and functional potential. Strains within the same species from SD104 were selected randomly. Another SMC (SMC-SD43), comprising all the species encompassed by sourdough SD43, was reconstructed with the aim of mimicking the potential of a spontaneous sourdough. The cell densities of all species used to reproduce the SMCs corresponded to those found in traditional spontaneous sourdoughs, as estimated by culturomics (Table 1). One experiment with these 2 SMCs involved their daily propagation at 30 °C for 30 days under in vivo sourdough conditions. Plate counts and isolation for RAPD-PCR profiling [34] were performed every 10 days. Analysis and comparison of biotypes were performed with BioNumerics software (v. 8.0, Applied Maths) using the reference profiles of each bacterial and yeast species belonging to the 2 SMCs. Metabolomics analyses allowed the characterization of volatile organic compounds (VOCs) after 1 and 30 days of propagation. To simulate the sourdough-like environment under sterile conditions, another experiment used WFH (wheat flour hydrolyzed) liquid medium [35]. The 2 SMCs were inoculated with 7 log cfu/mL of core dominant bacteria, 5 log cfu/mL of core subdominant bacteria and satellites, and 6 log cfu/mL of yeasts. The fermentation lasted for 12 h at 30 °C. With the aim of assessing how the elimination of microbial members might affect the transcriptomic profile of SDG, 7 SMCs were reconstructed by depleting the consortium 1 member at a time (SDG1 to SDG7). Acidification and growth kinetics were recorded using a benchtop online pH meter with a food probe (Hanna Instruments, Woonsocket, RI, USA) and a Tecan Infinite M NANO + spectrophotometer (Tecan Ltd., Switzerland). The growth kinetics were modeled according to the Gompertz Eq [36].. Metatranscriptomes were sequenced at exponential (6 h) and stationary (12 h) phases of growth. The bioinformatics pipeline described in the previous section (SqueezeMeta bioinformatics parameters and nested software) and the bioinformatics approach described above allowed the annotation and quantification of transcripts. An ad hoc reconstructed database allowed us to query the transcriptome project data and to extract per-species contributions.

**Table 1:** Composition of synthetic microbial communities (SMCs). De novo reconstructed Global sourdough (SDG) mimicking a natural sourdough from all omics data and SMC-SD43 mimicking the natural species composition of sourdough SD43. Final composition of SMC reporting the species, cell density found by culturomics in sourdoughs, their classification based on their proportions, and final number of included species

SDG				SMC-SD43			
**Taxa**	**Cell density (cfu mL**^**−1**^**)**	**Group**	**n. of species**	**Taxa**	**Cell density (cfu mL**^**−1**^**)**	**Group**	**n. of species**
**Lactic acid bacteria**				**Lactic acid bacteria**			
*Lactiplantibacillus plantarum*	9.96	Core dominant		*Lacticaseibacillus paracasei*	2.11	Core subdominant	
*Limosilactobacillus fermentum*	8.45	Core dominant		*Lactiplantibacillus plantarum*	6.48	Core dominant	
*Furfurilactobacillus rossiae*	5	Core subdominant	**4**	*Lacticaseibacillus rhamnosus*	2.48	Core subdominant	**7**
*Pediococcus pentosaceus*	3.23	Core subdominant		*Fructilactobacillus sanfranciscensis*	9.96	Core dominant	
				*Lactococcus lactis*	2.7	Core subdominant	
				*Leuconostoc citreum*	3.18	Core subdominant	
				*Weissella confusa*	8.48	Core dominant	
**Other bacteria**				**Other bacteria**			
*Staphylococcus epidermidis*	2.48	Satellite	**1**	*Staphylococcus sp.*	2	Satellite	**1**
**Yeasts**				**Yeasts**			
*Saccharomyces cerevisiae*	6.23	Core dominant	**2**	*Saccharomyces cerevisiae*	6.26	Core dominant	**1**
*Pichia kudriavzevii*	5.85	Dispensable dominant					

### Metabolic networking of sourdough core, dispensable and satellite microbial members

Based on the importance of sourdough performance, we selected the following metabolic mega-pathways: carbohydrate metabolism, pyruvate/energy production-conversion and nitrogen metabolism. Core enzymatic pathways, including enzymes detected in all sourdough metagenomes, were identified. In addition, dispensable/accessory genes encoding for the enzymatic portfolio among sourdough metagenomes were identified by inspecting the ad hoc build up sourdough SqueezeMeta database. KEGG abundance values (expressed as transcripts per million (TPM) values after normalizing for both sequencing depth and gene length) were used for a two-sample G-test (w/Yates’ + Fisher test) with Benjamini–Hochberg multiple test correction in STAMP (statistical analysis of taxonomic and functional profiles) software [37]. Metatranscriptomics was used together with metagenomics to find the active pathways within sourdoughs.

### GC–MS measurements

The VOC profile was analyzed by gas chromatography–mass spectrometry (GC–MS). A PAL COMBI-xt autosampler (CTC combiPAL, CTC Analysis AG, Zwingen, Switzerland) was used to standardize the headspace solid-phase microextraction (HS-SPME) procedure according to Liu et al. (2020) [38]. A Clarus 680 (Perkin Elmer, Beaconsfield, UK) gas chromatograph equipped with a Rtx-WAX column (30 m × 0.25 mm i.d., 0.25 μm film thickness) (Restek Superchrom, Milano, Italy) was used to thermally desorb and separate the headspace VOC [39]. Each chromatogram was analyzed for peak identification by comparing (i) the retention time (RT) of the detected compound with those of the provided pure standard for HPLC (Sigma–Aldrich, St. Louis, MO, USA) and (ii) experimental mass spectra with those of the National Institute of Standards and Technology database (NIST/EPA/NIH Mass Spectral Library with Search Program, data version NIST 05, software version 2.0d). A peak area threshold larger than 1 M and a match percentage higher than 85% were used as the criteria for identification. VOCs were quantified by using 2-methyl-4-pentanol (final concentration of 33 mg/l) as the internal standard.

### Data analysis

Metagenomics and metatranscriptomics data belonging to eight spontaneous sourdoughs and sixteen metatranscriptomics datasets from seven depleted SMCs plus one complete reconstructed SMC (at the exponential and stationary phases of growth) were analyzed. Two de novo reconstructed SMCs (SDG and SMC-SD43) were propagated for 30 days, and the single contributions of dominant, subdominant and satellite species were used to populate the subset of the investigated metabolic pathway functions. In turn, these data have been compared with those of the reconstructed sourdough (SDG1-SDG7). Statistically significant gene and transcript comparisons were obtained using an ANOVA Tukey–Kramer post hoc comparison test (corrected for multiple tests with the Benjamini–Hochberg procedure) in STAMP (statistical analysis of taxonomic and functional profiles) v2.1.3 software [37]. The input file matrices from the metagenomics and metatranscriptomics data and the relative metadata were customized to fit the STAMP software requirements. Culturomics analyses were carried out on 2 biological replicates for the same length of time (up to 30 days) as for VOC analyses.

## Results

### Sourdough richness in culturable bacteria and yeasts

Our isolation, purification and characterization procedures resulted in the creation of a sourdough biobank comprising a total of 1,661 isolates (1,488 bacteria and 173 yeasts). Spontaneous sourdoughs harbored presumptive lactic acid bacteria at ca. 2.0 to ca. 9.9 log cfu/g. Presumptive staphylococci and micrococci ranged from ca. 2.0 to ca. 7.0 log cfu/g. Presumptive coliforms were identified only in SD1 (ca. 2.5 log cfu/g) and SD69 (ca. 4.5 log cfu/g). All sourdoughs harbored ca. 6.5 log cfu/g yeasts. Partial sequencing of the 16S rRNA and 26S rRNA genes of all 1,661 isolates allowed the taxonomic identification of 36 species. The sourdough richness in culturable bacteria and yeast species varied from 8 (SD93) to 15 (SD44 and SD104), with differences in cell densities and prevalence. The sourdough species prevalence was calculated as the number of isolates identified per species divided by their total number. This percentage varied from 1 to 67%, mostly depending on the abundance level of each species (Table 2). Following this culturomics approach, the sourdough biobank also allowed us to obtain an overview of the ratio among players. Based on the correlation between back slopping steps and LAB cell density until sourdough reaches its maturity, we defined a species to be dominant or subdominant based on whether its cell density was greater or lower than 6 log cfu/g [40]. In addition, considering commensal interaction as a primary impacting factor, the natural selection of species until sourdough is mature is dependent on cell load and other technological parameters whose impacts can be even more important [40].

**Table 2:** Cultivable bacteria and yeasts identified in the eight spontaneous sourdoughs. Total number of isolates identified per sourdough, percentage of prevalence and cell density (log cfu/g)

Sourdough	Species	N° of isolates	Percentage of prevalence %	Cell density (log cfu/g)
	**Total all sourdoughs**	**1661**		
**SD1**	**Subtotal SD1**	**190**		
	**Lactic acid bacteria subtotal SD1**	**161**		
	*Lactiplantibacillus plantarum*	17	9	8.23
	*Lacticaseibacillus rhamnosus*	7	4	6.85
	*Fructilactobacillus sanfranciscensis*	128	67	9.97
	*Weissella confusa*	2	1	6.30
	*Leuconostoc citruem*	2	1	2.30
	*Enterococcus faecalis*	3	2	6.48
	*Lactococcus lactis*	2	1	2.30
	**Other bacteria subtotal SD1**	**4**		
	*Staphylococcus sp.*	2	1	2.30
	*Enterobacter sp.*	2	1	2.30
	**Yeasts subtotal SD1**	**25**		
	*Saccharomyces cerevisiae*	23	12	6.36
	*Saccharomyces sp. / Naumovozyma castellii*	2	1	5.30
**SD43**	**Subtotal SD43**	**163**		
	**Lactic acid bacteria subtotal SD43**	**147**		
	*Lacticaseibacillus paracasei*	3	2	2.11
	*Lactiplantibacillus plantarum*	20	12	6.48
	*Fructilactobacillus sanfranciscensis*	98	60	9.96
	*Lacticaseibacillus rhamnosus*	3	2	2.48
	*Lactococcus lactis*	5	3	2.70
	*Leuconostoc citreum*	15	9	3.18
	*Weissella confusa*	3	2	8.48
	**Other bacteria subtotal SD43**	**1**		
	*Staphylococcus sp.*	1	1	2.00
	**Yeasts subtotal SD43**	**15**		
	*Saccharomyces cerevisiae*	15	9	6.26
**SD44**	**Subtotal SD44**	**234**		
	**Lactic acid bacteria subtotal SD44**	**206**		
	*Lactiplantibacillus plantarum /Lactiplantibacillus pentosus*	35	15	7.54
	*Limosilactobacillus fermentum*	7	3	6.85
	*Latilactobacillus curvatus*	8	3	6.95
	*Leuconostoc citreum*	36	15	7.56
	*Leuconostoc mesenteroides*	2	1	6.00
	*Leuconostoc pseudomesenteroides*	7	3	6.85
	*Weissella cibaria*	45	19	7.67
	*Weissella confusa*	39	17	7.59
	*Pediococcus pentosaceus*	17	7	3.23
	*Enterococcus sp.*	4	2	2.60
	*Enterococcus faecalis*	2	1	2.30
	*Enterococcus faecium*	4	2	2.60
	**Other bacteria subtotal SD44**	**4**		
	*Staphylococcus sp.*	2	1	2.30
	*Staphylococcus aureus*	2	1	2.00
	**Yeasts subtotal SD44**	**24**		
	*Saccharomyces cerevisiae*	24	10	6.15
**SD69**	**Subtotal SD69**	**186**		
	**Lactic acid bacteria subtotal SD69**	**162**		
	*Lactiplantibacillus plantarum*	60	32	8.78
	*Fructilactobacillus sanfranciscensis*	60	32	7.88
	*Lacticaseibacillus rhamnosus*	5	3	4.70
	*Furfurilactobacillus rossiae*	2	1	2.00
	*Lactococcus lactis*	25	13	6.36
	*Leuconostoc citreum*	6	3	4.78
	*Leuconostoc sp.*	4	2	6.60
	**Other bacteria subtotal SD69**	**6**		
	*Enterobacter sp.*	3	2	4.48
	*Staphylococcus warneri / Staphylococcus pasteuri*	1	1	2.00
	*Staphylococcus epidermidis*	2	1	2.30
	**Yeasts subtotal SD69**	**18**		
	*Saccharomyces cerevisiae*	18	10	6.23
**SD88**	**Subtotal SD88**	**229**		
	**Lactic acid bacteria subtotal SD88**	**195**		
	*Lactiplantibacillus plantarum*	140	61	8.16
	*Lactiplantibacillus pentosus*	5	2	6.70
	*Lacticaseibacillus rhamnosus*	4	2	5.60
	*Lactobacillus sp.*	2	1	2.00
	*Weissella confusa*	4	2	6.60
	*Leuconostoc citreum*	30	13	7.43
	*Enterococcus sp.*	5	2	2.70
	*Enterococcus faecium*	5	2	2.70
	**Other bacteria subtotal SD88**	**4**		
	*Staphylococcus aureus*	2	1	2.00
	*Staphylococcus epidermidis / Staphylococcus caprae*	2	1	2.30
	**Yeasts subtotal SD88**	**30**		
	*Pichia kudriavzevii*	30	13	6.48
**SD93**	**Subtotal SD93**	**205**		
	**Lactic acid bacteria subtotal SD93**	**182**		
	*Lactiplantibacillus plantarum*	24	12	8.32
	*Limosilactobacillus fermentum*	90	44	8.94
	*Leuconostoc citreum*	43	21	7.63
	*Weissella confusa*	14	7	7.15
	*Enterococcus faecium / Enterococcus durans*	9	4	2.95
	*Enterococcus faecalis*	2	1	2.30
	**Other bacteria subtotal SD93**	**3**		
	*Staphylococcus epidermidis*	3	1	2.48
	**Yeasts subtotal SD93**	**20**		
	*Saccharomyces cerevisiae*	20	10	6.30
**SD102**	**Subtotal SD102**	**234**		
	**Lactic acid bacteria subtotal SD102**	**209**		
	*Lactiplantibacillus plantarum*	51	22	8.66
	*Limosilactobacillus fermentum*	6	3	6.78
	*Fructilactobacillus sanfranciscensis*	3	1	5.00
	*Leuconostoc citreum*	99	42	9.00
	*Weissella confusa*	45	19	8.53
	*Enterococcus faecium*	5	2	2.70
	**Other bacteria subtotal SD102**	**5**		
	*Staphylococcus warneri*	3	1	2.48
	*Staphylococcus epidermidis / Staphylococcus caprae*	2	1	2.00
	**Yeasts subtotal SD102**	**20**		
	*Saccharomyces cerevisiae*	20	9	6.30
**SD104**	**Subtotal SD104**	**220**		
	**Lactic acid bacteria subtotal SD104**	**189**		
	*Lactiplantibacillus plantarum*	98	45	9.96
	*Fructilactobacillus sanfranciscensis*	32	15	9.41
	*Limosilactobacillus fermentum*	29	13	8.45
	*Lacticaseibacillus paracasei /Lacticaseibacillus rhamnosus*	5	2	6.70
	*Furfurilactobacillus rossiae*	2	1	6.00
	*Lacticaseibacillus rhamnosus*	2	1	3.00
	*Weissella confusa*	10	5	7.00
	*Leuconostoc citreum*	3	1	6.48
	*Enterococcus faecalis*	8	4	2.85
	**Other bacteria subtotal SD104**	**10**		
	*Staphylococcus epidermidis / Staphylococcus capitis*	7	3	6.85
	*Staphylococcus epidermidis / Staphylococcus caprae*	3	1	1.48
	**Yeasts subtotal SD104**	**21**		
	*Saccharomyces cerevisiae*	12	5	6.08
	*Pichia kudriavzevii*	7	3	5.85
	*Pichia sp.*	2	1	5.30

Moreover, we consider satellite species to be those nonlactic acid bacteria that are occasionally found in sourdough. *Lac. plantarum* was dominant or codominant in all sourdoughs. *Leuconostoc citreum* was also identified in all sourdoughs, with cell densities varying from ca. 3.2 to 9.0 log cfu/g. Except for SD1*,* enterococci were present at low cell density (ca. 2 log cfu/g) only in some sourdoughs. Except for SD104, staphylococcal species were mostly identified as satellite members. *Sac. cerevisiae* was the dominant yeast found in all spontaneous sourdoughs, with cell densities ranging from 6.1 to 6.4 log cfu/g. The only exception was SD88, which harbored *Pic. kudriavzevii* (ca. 6.5 log cfu/g).

### Sourdough richness in bacteria and yeasts as estimated by metagenomics

**Fig. 2:**
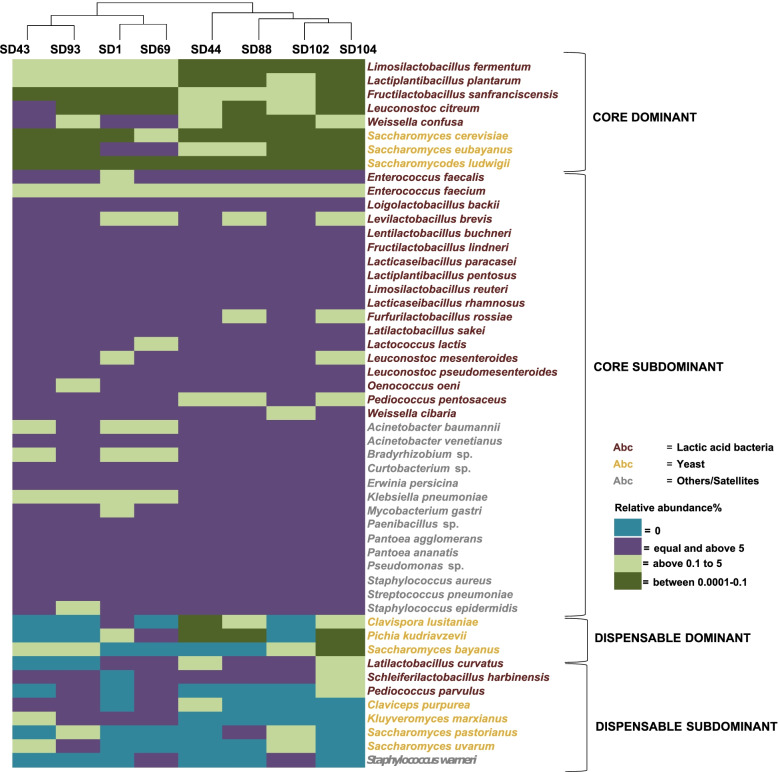
Core and dispensable species found in the 8 spontaneous sourdoughs by shotgun metagenomics. Pseudoheatmap displaying the core (OTUs shared by the eight sourdoughs) and dispensable (OTUs variously detected in at least one sourdough) microbiomes under sourdough conditions. Dominant species are defined with a relative abundance of ≥ 5% of the total bacterial metagenome in at least one sourdough and are subdominant below. Samples are clustered at the top based on species relative abundances using the Euclidean distance. The color bar on the right describes the species prevalence

Approximately 6.9 M contigs, for a total of 2.8 Gbp, led to the assembly combining all sourdoughs (Supplementary Table S2, Additional File 2). The longest contig was ca. 245 Kbp. The assembled N50 and N90 values were 813 and 516 base pairs, respectively. Looking at the rank assignation for the whole assembly, 195, 333 and 385 contigs were assigned to family, genus and species ranks, respectively. Among prokaryotes, sourdoughs harbored 202 genera and 275 species (Supplementary Table S3, Additional File 3). Eukaryotic taxa (yeasts) accounted for 30 genera and 31 species (Supplementary Table S4, Additional File 4). The high biodiversity we detected was ultimately reflected in the species relative abundances, ranging from 0.0001 to 80%. For metagenomics data, considering those species that were present in at least one sourdough, we defined dominant or subdominant species based on whether their percentage of relative abundance was greater or lower than 5%, as normalized to the total metagenome content. Similarly, in other metagenomics studies, dominance/subdominance was defined based on species richness and relative abundance level [41, 42]. Regardless of the relative abundance, we defined core or dispensable species according to whether they were shared by all sourdoughs or not (Fig. 2). Within the core microbiome set of species, *Lac. plantarum*, *Fru. sanfranciscensis*, *Lim. fermentum*, *Leuc. citreum* and *Weissella confusa* were dominant in 1 or more sourdoughs, which agreed with culturomics data (Table 2). Eighteen subdominant species of lactic acid bacteria were also found. The core sourdough microbiome also harbored other bacterial species. The core yeast microbiome (*Saccharomycodes ludwigii* and *Sac. cerevisiae*) was dominant in all sourdoughs except for SD1 and SD69, in which *Saccharomyces eubayanus* was dominant. Dispensable dominant yeasts included *Pichia kudriavzevii*, *Clavispora lusitaniae* and *Saccharomyces bayanus*, which were shared by 5 and 4 sourdoughs, respectively (Supplementary Figure S1, Additional File 5).

### Inspecting gene annotation and deciphering the functional redundancy of sourdough genomes

To decipher the extent of functional redundancy in sourdough genomes, we retrieved the gene abundances in each sample by mapping a single set of reads (from each individual sample) against the total assembly obtained by pooling reads from all samples in a single bin (Supplementary Table S5, Additional File 6). The highest mapping percentage (84.47) was found for SD43. To detect missing genes and correct errors in gene prediction, we used a double-pass step procedure named “extrasensitive ORF prediction”, which consisted of gene prediction as a first step and BLASTX search as a second step.

The total predicted ORF number was nonhomogeneous (Supplementary Table S6, Additional File 7). Considering the Prodigal results, SD1, SD69 and SD93 showed comparable numbers (over 1 M ORFs, with SD1 having a peak of approximately 2 M ORFs). SD43, SD44, SD88, SD102 and SD104 exhibited fewer than 1 M total predicted ORFs. On the other hand, the number of predicted KEGG functions was more homogeneously distributed, with the lowest and highest numbers (ca. 61 K and 551 K) found in SD102 and SD104, respectively. The set of KEGG functions shared among all spontaneous sourdoughs (core metagenome) spanned 1163 genes (Supplementary Table S7, Additional File 8). The other accessory genes were heterogeneously distributed. A set of unique genes was found in each sourdough, ranging from 33 (SD43) to 684 (SD1) unique genes. Adhering to the concept that housekeeping gene functions were always maintained by every species, sourdoughs with a higher number of species showed a much higher number of copies per pathway for these functions (Supplementary Table S8, Additional File 9). The requirements for fermentation, in terms of biochemical pathways, for all sourdoughs are related to carbohydrates, pyruvate and energy production and conversion, nitrogen (proteolytic systems, transport and catabolism of amino acids) and stress response. The majority of genes that we found in our sourdough metagenomics data are related to these main pathways.

### Players actively engaged in sourdough metabolism

With the aim of identifying metabolically active species, we performed a metatranscriptomics experiment. Transcript profiles and comparison with metagenomics data were performed only for those species with a relative abundance higher than 0.1% in at least 1 sourdough. As a result, all metagenomics data from *Lactobacillus* (former taxonomy) and *Weissella* species, were confirmed in terms of taxa viability and relative expression profiles by culturomics and metatranscriptomics experiments (Fig. 2). Notably, species of *Lactococcus* and *Enterococcus,* as identified by metagenomics, were culturable but not transcriptionally active. Moreover, all *Leuconostoc*, *Oenococcus* and *Pediococcus* species identified by metagenomics were transcriptionally active, and almost all were culturable. Conversely, most of the other nonlactic acid bacteria were transcriptionally inactive and nonculturable. *Staphylococcus aureus, Staphylococcus epidermidis* and *Staphylococcus warneri* were culturable but transcriptionally inactive. Except in SD88, *Sac. cerevisiae* was always transcriptionally active and cultivable. Some yeast species were transcriptionally inactive and/or nonculturable (Fig. 2).

### Reconstruction of the main metabolic pathways underlying player functionality

We aimed to capture the potential contribution of those players involved in the metabolic pathways that have a key role in sourdough functionality and thus impact resilience and stability. The investigation of genes and transcripts related to the main pathways relevant to sourdough biotechnological properties allowed us to determine the contributions of species belonging to dominant, subdominant and satellite groups (Supplementary Figure S2, Additional File 10). Starch, nonstarch polysaccharide and sucrose pathway core genes (shared by all sourdoughs), together with those heterogeneously represented by a few species (accessory genes), are listed in Table 3. More specifically, higher gene and transcript numbers were harbored by core dominant species, such as *Fru. sanfranciscensis*, *Lim. fermentum*, *Lac. plantarum, Leuc. citreum* and *Weissella confusa* and *Sac. cerevisiae*, whereas subdominant and dispensable species contributed fewer genes and transcripts.

**Table 3:** Distribution of gene encoding for metabolism of starch and sucrose in the metagenome of eight spontaneous sourdoughs. The expressed genes, evaluated through meta-tascriptomic analyses, have been marked by double asterisk

	Genes	Dominant							Sub-dominant			
		Fru. sanfranciscensis	Lim. fermentum	Lac. plantarum	Leu. citreum	Wes. confusa	Sac. cerevisiae	Pic. kudriavzevii	Lac. paracasei	Lev. brevis	Lat. curvatus	Fur. rossiae
CORE GENES	1,3-beta-glucan synthase [EC:2.4.1.34]						**	**				
	6-phospho-beta-glucosidase [EC:3.2.1.86]			**	**	*			*	*	*	
	alpha-glucosidase [EC:3.2.1.20]	**		**		**			*	**	*	*
	alpha,alpha-trehalase [EC:3.2.1.28]			*			**	**				
	beta-fructofuranosidase [EC:3.2.1.26]		**	**	**	**	**		*			
	beta-phosphoglucomutase [EC:5.4.2.6]	**	**	**	**	**				**	*	**
	dextransucrase [EC:2.4.1.5]	**	**		**	**						
	fructokinase [EC:2.7.1.4]	**	**	**	**	*					*	*
	glucan 1,3-beta-glucosidase [EC:3.2.1.58]						**	**				
	glucokinase [EC:2.7.1.2]	**	**	**	**	**			*	**	*	*
	glucose-6-phosphate isomerase [EC:5.3.1.9]	**	**	*	**		**	**				*
	glycogen debranching enzyme [EC:2.4.1.25 3.2.1.33]						**	**				
	glycogen phosphorylase [EC:2.4.1.1]			**		*	**	**	*			
	glycogen synthase [EC:2.4.1.11]						**	**				
	hexokinase [EC:2.7.1.1]						**	**				
	maltose phosphorylase [EC:2.4.1.8]	**	**	**	**	**			*	**	**	**
	oligo-1,6-glucosidase [EC:3.2.1.10]	*	**	**	**		**		*	*		*
	phosphoglucomutase [EC:5.4.2.2]	**	**	**	*	*	**	**		**		
	PTS system, cellobiose-specific IIA component [EC:2.7.1.196 2.7.1.205]			**	**	*			*		*	
	PTS system, cellobiose-specific IIB component [EC:2.7.1.196 2.7.1.205]			**	**	*				*		
	PTS system, cellobiose-specific IIC component		**	**	**	**			*	**	*	**
	trehalose 6-phosphate synthase [EC:2.4.1.15 2.4.1.347]						**	**				
	trehalose-6-phosphate hydrolase [EC:3.2.1.93]	*		**	*						*	
	UTP--glucose-1-phosphate uridylyltransferase [EC:2.7.7.9]	**	**	*	*		**	**		*		**
ACCESSORY GENES	1,4-alpha-glucan branching enzyme [EC:2.4.1.18]			**			**					
	2,4-alpha-glucan branching enzyme [EC:2.4.1.18]								*			
	3,4-alpha-glucan branching enzyme [EC:2.4.1.18]					*						
	alpha-amylase [EC:3.2.1.1]			**								
	alpha,alpha-trehalose phosphorylase [EC:2.4.1.64]			**						**		**
	beta-glucosidase [EC:3.2.1.21]					*			*	**	*	*
	cellulose synthase (UDP-forming) [EC:2.4.1.12]	*		**	**							*
	cyclomaltodextrinase / maltogenic alpha-amylase / neopullulanase [EC:3.2.1.54 3.2.1.133 3.2.1.135]			**		**			*		*	
	ectonucleotide pyrophosphatase/phosphodiesterase family member 1/3 [EC:3.1.4.1 3.6.1.9]						**					
	endoglucanase [EC:3.2.1.4]						**			*		
	glucoamylase [EC:3.2.1.3]						**					
	glucose-1-phosphate adenylyltransferase [EC:2.7.7.27]			**		*						
	inulosucrase [EC:2.4.1.9]	*										
	maltose-6'-phosphate glucosidase [EC:3.2.1.122]		*	**								
	PTS system, sugar-specific IIA component [EC:2.7.1.-]			**							*	
	starch synthase [EC:2.4.1.21]			**		*						
	sucrose phosphorylase [EC:2.4.1.7]		*		*							
	trehalose 6-phosphate synthase complex regulatory subunit						**	**				
	trehalose 6-phosphate synthase/phosphatase [EC:2.4.1.15 3.1.3.12]						**	**				

	Genes	Sub-dominant									Satellites
		Lim. reuteri	Lac. sakey	Lac. rhamnosus	Ped. pentosaceus	Ped. parvulus	Leu. mesenteroides	Leu. pseudomesenteroides	Leu. sp	Lac.lactis	Erw. persicina
CORE GENES	1,3-beta-glucan synthase [EC:2.4.1.34]										
	6-phospho-beta-glucosidase [EC:3.2.1.86]				**	*	*	*			
	alpha-glucosidase [EC:3.2.1.20]			*		*					
	alpha,alpha-trehalase [EC:3.2.1.28]										
	beta-fructofuranosidase [EC:3.2.1.26]						*	**			
	beta-phosphoglucomutase [EC:5.4.2.6]				**		*				
	dextransucrase [EC:2.4.1.5]	*					*	*			
	fructokinase [EC:2.7.1.4]		*		**						
	glucan 1,3-beta-glucosidase [EC:3.2.1.58]						*		**		
	glucokinase [EC:2.7.1.2]				**	*	*			*	
	glucose-6-phosphate isomerase [EC:5.3.1.9]				*						
	glycogen debranching enzyme [EC:2.4.1.25 3.2.1.33]										
	glycogen phosphorylase [EC:2.4.1.1]			*							
	glycogen synthase [EC:2.4.1.11]										
	hexokinase [EC:2.7.1.1]										
	maltose phosphorylase [EC:2.4.1.8]				**	*	*				
	oligo-1,6-glucosidase [EC:3.2.1.10]					*	*	**			
	phosphoglucomutase [EC:5.4.2.2]				**	*	*				
	PTS system, cellobiose-specific IIA component [EC:2.7.1.196 2.7.1.205]				**		*				
	PTS system, cellobiose-specific IIB component [EC:2.7.1.196 2.7.1.205]				**		*				
	PTS system, cellobiose-specific IIC component				**	*	*	*		*	
	trehalose 6-phosphate synthase [EC:2.4.1.15 2.4.1.347]										
	trehalose-6-phosphate hydrolase [EC:3.2.1.93]				**	*	*				
	UTP--glucose-1-phosphate uridylyltransferase [EC:2.7.7.9]				*						
ACCESSORY GENES	1,4-alpha-glucan branching enzyme [EC:2.4.1.18]										
	2,4-alpha-glucan branching enzyme [EC:2.4.1.18]										
	3,4-alpha-glucan branching enzyme [EC:2.4.1.18]										
	alpha-amylase [EC:3.2.1.1]					*					
	alpha,alpha-trehalose phosphorylase [EC:2.4.1.64]										
	beta-glucosidase [EC:3.2.1.21]						*	**			
	cellulose synthase (UDP-forming) [EC:2.4.1.12]				**		*				*
	cyclomaltodextrinase / maltogenic alpha-amylase / neopullulanase [EC:3.2.1.54 3.2.1.133 3.2.1.135]									*	
	ectonucleotide pyrophosphatase/phosphodiesterase family member 1/3 [EC:3.1.4.1 3.6.1.9]										
	endoglucanase [EC:3.2.1.4]						*				
	glucoamylase [EC:3.2.1.3]										
	glucose-1-phosphate adenylyltransferase [EC:2.7.7.27]										
	inulosucrase [EC:2.4.1.9]						*				
	maltose-6'-phosphate glucosidase [EC:3.2.1.122]										
	PTS system, sugar-specific IIA component [EC:2.7.1.-]				*	*					
	starch synthase [EC:2.4.1.21]										
	sucrose phosphorylase [EC:2.4.1.7]						*				
	trehalose 6-phosphate synthase complex regulatory subunit										
	trehalose 6-phosphate synthase/phosphatase [EC:2.4.1.15 3.1.3.12]										

The pathways involved in hexose fermentation (Embden-Meyerhof-Parnas and phosphoketolase pathways), pentose metabolism (phosphoketolase pathways), and the alternative fate of pyruvate are shown in Table 4.. Additionally, in this case, the selected core metagenome encoded all transcripts needed to ferment hexoses and pentoses (Table 4.). With few exceptions, all the basic functions at both the genomic and transcriptomic levels are performed by the dominant species. Among subdominant species, most of the genes for the pentose phosphate pathway were found in *Lev. brevis, Fur. rossiae, Ped. pentosaceus*, and/or *Pic. kudriavzevii* genomes. Key genes, such as transaldolase (EC:2.2.1.2) and transketolase (EC:2.2.1.1), were detectable in all sourdoughs. The genes encoding enzymes to synthesize acetate were detected in the dominant species of each sourdough, except for *Fru. sanfranciscensis*. The acetate pathway was also present in subdominant species *Fur. rossiae, Lev brevis, Ped. pentosaceus* and *Leu. mesenteroides*. Some strains of *Sac. cerevisiae* harbored the gene encoding acetyl-CoA synthetase (EC:6.2.1.1). This activity was found in all sourdoughs except for SD102. Additionally, for this pathway, the core dominant group contributed the highest number of genes and relative transcripts (Table 4.).

**Table 4:** Distribution of gene encoding for metabolism of pentose phosphate pathway in the metagenome of the eight spontaneous sourdoughs. The expressed genes, evaluated through meta-tascriptomic analyses, were marked by bouble asterisk

	Genes	Dominant							Sub-dominant		
		Fru. sanfranciscensis	Lac. plantarum	Lim. fermentum	Leuc. citreum	Wei. confusa	Sac. cerevisiae	Pic. kudriavzevii	Lev. brevis	Fur. rossiae	Lat. curvatus
CORE GENES	3-hexulose-6-phosphate synthase [EC:4.1.2.43]	*	*				**		*		
	6-phosphofructokinase 1 [EC:2.7.1.11]		*				**				
	6-phosphogluconate dehydrogenase [EC:1.1.1.44 1.1.1.343]	**	**	**	**	*	**	**	*	**	*
	6-phosphogluconolactonase [EC:3.1.1.31]	**	**		**		**	**	**		*
	fructose-bisphosphate aldolase, class II [EC:4.1.2.13]	*	**			*	**	**	*		*
	gluconokinase [EC:2.7.1.12]	**	**	**	*	**	**		*	*	*
	glucose-6-phosphate 1-dehydrogenase [EC:1.1.1.49 1.1.1.363]	**	**	**	**	*	**		*	*	
	glucose-6-phosphate isomerase [EC:5.3.1.9]	**	*	**	**		**	**		*	
	phosphoglucomutase [EC:5.4.2.2]	**	**	**	*	*	**	**	**		
	phosphopentomutase [EC:5.4.2.7]	**		**					**	*	
	ribokinase [EC:2.7.1.15]	**	**	**	**	*	**	**		*	
	ribose 5-phosphate isomerase A [EC:5.3.1.6]	**	**	**	**	**	*	**	**	*	
	ribose-phosphate pyrophosphokinase [EC:2.7.6.1]	**	**	**	**	**	**	**	*	**	
	ribulose-phosphate 3-epimerase [EC:5.1.3.1]	**	*	**	**		*	**	*	**	
	transaldolase [EC:2.2.1.2]		**	**			*	**	**	*	
	transketolase [EC:2.2.1.1]	*	**	**	**		**	**	*	*	
	xylulose-5-phosphate/fructose-6-phosphate phosphoketolase [EC:4.1.2.9 4.1.2.22]	**	**	**	*	*				*	
ACCESSORY GENES	4-hexulose-6-phosphate synthase [EC:4.1.2.43]									*	
	6-phospho-3-hexuloisomerase [EC:5.3.1.27]								*		
	10-phosphogluconolactonase [EC:3.1.1.31]									*	
	11-phosphogluconolactonase [EC:3.1.1.31]					*					
	2-dehydro-3-deoxy-phosphogluconate aldolase [EC:4.1.2.14]		**								
	2-dehydro-3-deoxygluconokinase [EC:2.7.1.45]		**		**				*		
	2-dehydro-3-deoxyphosphogluconate aldolase / (4S)-4-hydroxy-2-oxoglutarate aldolase [EC:4.1.2.14 4.1.3.42]		*						*		
	7-phosphogluconolactonase [EC:3.1.1.31]			**							
	8-phosphogluconolactonase [EC:3.1.1.31]									**	
	9-phosphogluconolactonase [EC:3.1.1.31]			*							
	deoxyribose-phosphate aldolase [EC:4.1.2.4]			**					*		*
	fructose-1,6-bisphosphatase I [EC:3.1.3.11]							**			
	fructose-1,6-bisphosphatase III [EC:3.1.3.11]		**								*
	glucose 1-dehydrogenase [EC:1.1.1.47]		*	*	**	*					**

	Genes	Sub-dominant									Satellites
		Lac. paracasei	Lac. rhamnosus	Fru. lindneri	Leu.mesenteroides	Leu.pseudomesenteroides	Ped. pentosaceus	Ped. parvulus	Lac.lactis	Ent. faecium	Erw. persicina
CORE GENES	3-hexulose-6-phosphate synthase [EC:4.1.2.43]						**				
	6-phosphofructokinase 1 [EC:2.7.1.11]						*				
	6-phosphogluconate dehydrogenase [EC:1.1.1.44 1.1.1.343]				*		**		*	*	
	6-phosphogluconolactonase [EC:3.1.1.31]				*		**	*			
	fructose-bisphosphate aldolase, class II [EC:4.1.2.13]		*				*				
	gluconokinase [EC:2.7.1.12]	*	*				**	*			
	glucose-6-phosphate 1-dehydrogenase [EC:1.1.1.49 1.1.1.363]	*					**				
	glucose-6-phosphate isomerase [EC:5.3.1.9]						*				
	phosphoglucomutase [EC:5.4.2.2]				*		**	*			
	phosphopentomutase [EC:5.4.2.7]	*									
	ribokinase [EC:2.7.1.15]	*			*	*	*				*
	ribose 5-phosphate isomerase A [EC:5.3.1.6]				*		**		*		
	ribose-phosphate pyrophosphokinase [EC:2.7.6.1]						**				
	ribulose-phosphate 3-epimerase [EC:5.1.3.1]						**				
	transaldolase [EC:2.2.1.2]										
	transketolase [EC:2.2.1.1]		*								
	xylulose-5-phosphate/fructose-6-phosphate phosphoketolase [EC:4.1.2.9 4.1.2.22]	*			*		*			*	
ACCESSORY GENES	4-hexulose-6-phosphate synthase [EC:4.1.2.43]										
	6-phospho-3-hexuloisomerase [EC:5.3.1.27]			*			**				
	10-phosphogluconolactonase [EC:3.1.1.31]										
	11-phosphogluconolactonase [EC:3.1.1.31]										
	2-dehydro-3-deoxy-phosphogluconate aldolase [EC:4.1.2.14]										
	2-dehydro-3-deoxygluconokinase [EC:2.7.1.45]		**		*		**				
	2-dehydro-3-deoxyphosphogluconate aldolase / (4S)-4-hydroxy-2-oxoglutarate aldolase [EC:4.1.2.14 4.1.3.42]		*								
	7-phosphogluconolactonase [EC:3.1.1.31]										
	8-phosphogluconolactonase [EC:3.1.1.31]										
	9-phosphogluconolactonase [EC:3.1.1.31]										
	deoxyribose-phosphate aldolase [EC:4.1.2.4]		**						*		
	fructose-1,6-bisphosphatase I [EC:3.1.3.11]										
	fructose-1,6-bisphosphatase III [EC:3.1.3.11]	*					**				
	glucose 1-dehydrogenase [EC:1.1.1.47]						*	*			

All sourdough metagenomes contained the bacterial and yeast genes responsible for peptide catabolism (Supplementary Table S9, Additional File 11). As evidenced by the species abundance in each sourdough, gene redundancy markedly varied in terms of transcript number. The core metagenome comprises a pattern of peptidases with different substrate specificities, such as 5 endopeptidases, 4 aminopeptidases, a tripeptide aminopeptidase and 3 dipeptidases, whose genes were expressed in all sourdoughs. The only exception was found for lactocepin EC:3.4.21.96. The core metagenome of all sourdoughs also harbored several proline-specific peptidases. Various other accessory peptidases were identified. Overall, *Sac. cerevisiae* and *Pic. kudriavzevii* have few proline-associated peptidases. The genes and transcripts relevant to the catabolism of branched chain amino acids (BCAAs), aromatic amino acids (ArAs) and free amino acids (FAAs) are listed (Supplementary Table S10, Additional File 12). Core dominants showed the highest gene and transcript contributions. The branched chain amino transferase (BcaT EC:2.6.1.42) was encoded by *Lac. plantarum*, *Lim. fermentum*, *Leuc. citreum* and one yeast (*Sac. cerevisiae*). Aminotransferases for aromatic amino acids (ArAT EC:2.6.1.57, EC:2.6.1.58 and EC:2.6.1.1) were only related to *Sac. cerevisiae* and/or *Pic. kudriavzevii*. Genes encoding L-asparaginase (EC:3.5.1.1) were widely distributed and active within the genome of dominant and subdominant lactic acid bacteria. Genes encoding arginine deiminase [EC:3.5.3.6], which is relevant to arginine degradation, were found to be active within the genome of *Lac. plantarum*, *Leuc. citreum*, *Wei. confusa* and *Ped. pentosaceus*.

### De novo design of synthetic microbial communities

We de novo reconstructed a synthetic microbial community (Sourdough Global, SDG) by merging the results from all meta-omics. The biobank was used as the resource of the dominant and subdominant species, which were eligible to be included if they harbored at least 20 key genes and transcripts for each of the above pathways and were present in 4 out of 8 spontaneous sourdoughs. Satellite members were selected based on their frequency of isolation[5]. The SDG comprised 7 species (Table 1). The factorial approach we used allowed us to investigate whether the depletion of one species at a time affected the functions of the remaining members, implying sourdough performance and stability.

### Players contributing to the metabolic resilience of de novo SDG

**Fig. 3:**
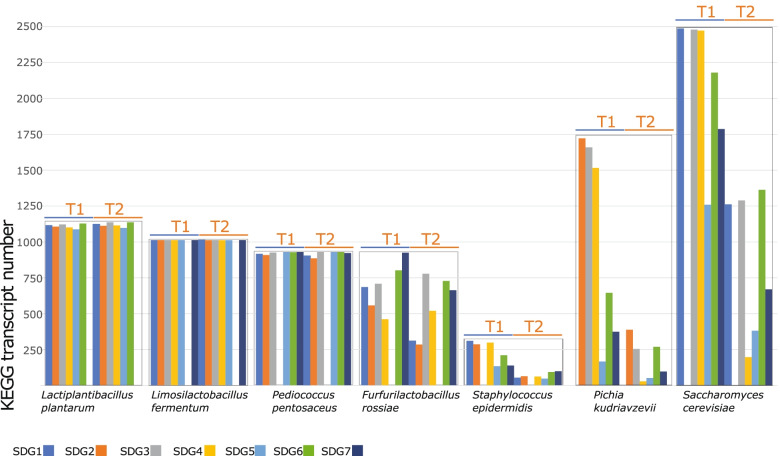
Total transcript KEGG function as bar plot histograms. Per-species contribution in terms of KEGG transcript presence/absence in both the exponential (T1) and stationary (T2) phases within each depleted synthetic microbial community (SMC-SDG1 without Pichia kudriavzevii; SMC-SDG2 without Saccharomyces cerevisiae, SMC-SDG3 without Staphylococcus epidermidis, SMC-SDG4 without Pediococcus pentosaceus, SMC-SDG5 without Furfurilactobacillus rossiae, SMC-SDG6 without Limosilactobacillus fermentum and SMC-SDG7 without Lactiplantibacillus plantarum)

The dominant KEGG function number for *Lac. plantarum* and *Lim. fermentum* did not vary between the exponential and stationary phases of growth, and it was not influenced by the depletion of a single species within the SDG (Fig. 3). Although the absolute value of KEGG transcripts was lower than that of the dominant species, the same stability was also observed for the subdominant *Ped. pentosaceus*. In contrast, the KEGG numbers for other bacteria and yeasts decreased upon reaching the stationary phase of growth and showed differential expression depending on the SDG composition. Species interactions modified the KEGG profiles when any single species was removed from the pools. The number of KEGG functions of the subdominant *Fur. rossiae* was driven by dominant species: the highest number of transcripts was observed in the pool without *Lim. fermentum* and *Lac. plantarum*. The opposite trend was observed during the stationary phase of growth without *Sta. epidermidis* or *Ped. pentosaceus*. The satellite *Sta. epidermidis* had the lowest KEGG transcript number when *Fur. rossiae* was absent and, more generally, showed a decreased number of KEGG functions when entering the stationary phase of growth. Nevertheless, its contribution during the exponential phase of growth was not negligible. The core dominant *Sac. cerevisiae* and the dispensable dominant *Pic. kudriavzevii* had the highest number of KEGG transcripts during the exponential phase of growth and when *Sta. epidermidis* and *Ped. pentosaceus* were absent (Fig. 3). *Sac. cerevisiae* had a higher number of transcripts than *Pic. kudriavzevii* in all SMCs.

**Fig. 4:**
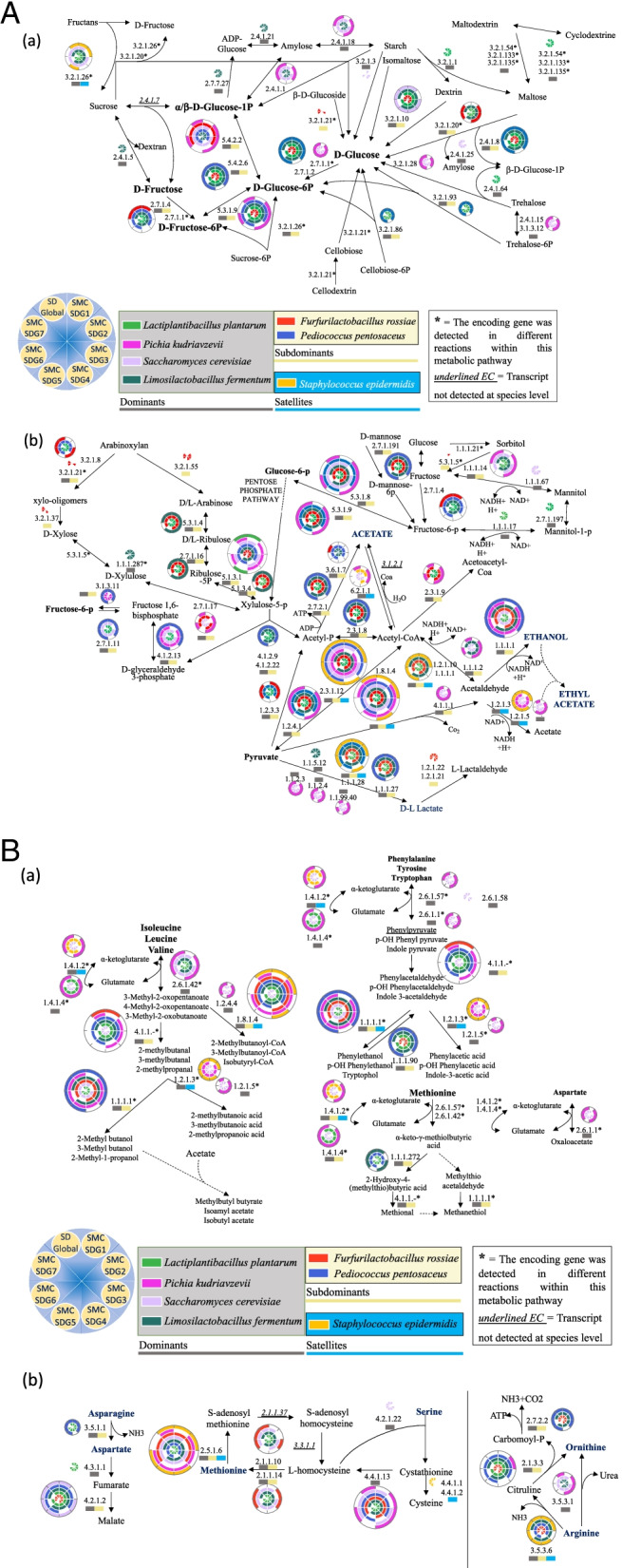
Reconstruction of carbohydrate and pyruvate pathways in SMCs. Schematic representation of metabolic pathways, according to the KEGG database, involved in a) carbohydrate metabolism, b) pentose and pyruvate metabolism, c) aminotransferases and c) deaminases and lyases for each synthetic microbial community (SDG, SMC-SDG1 without Pichia kudriavzevii; SMC-SDG2 without Saccharomyces cerevisiae, SMC-SDG3 without Staphylococcus epidermidis, SMC-SDG4 without Pediococcus pentosaceus, SMC-SDG5 without Furfurilactobacillus rossiae, SMC-SDG6 without Limosilactobacillus fermentum and SMC-SDG7 without Lactiplantibacillus plantarum) reconstructed based on metatranscriptomics data. Each species presence/absence contribution of a single enzymatic (EC number) reaction is reported in each concentric circle, wherein each slice refers to a single SMC and each color to a different species. The color flag underneath each EC number refers to the classification of species as dominants, subdominants and satellites. The circular diagram (bottom left) explains the SMC placement within circles

The SDG had the capability to mimic all the functions needed to express carbohydrate metabolism. The potential contribution of each species to the starch, nonstarch polysaccharide and sucrose pathways was reconstructed (Fig. 4a, b). Key enzymes for starch, cyclodextrin and maltodextrin hydrolysis were encoded by core and dispensable dominant species of SDG. *Lac. plantarum* was the only species involved in the pathways leading to maltose formation (e.g., alpha-amylase, EC:3.2.1.1 and cyclomaltodextrinase/neopullulanase, EC:3.2.1.54), while the other two core dominant species (*Lim. fermentum* and *Sac. cerevisiae*) showed the ability to modify the starch/amylose structure by releasing D-glucose-1P. Concomitant activity on the D-glucose-1-P pathway was also detected for the dispensable dominant *Pic. kudriavzevii*.

*Sac. cerevisiae* was the only species capable of directly hydrolyzing starch in D-glucose (glucoamylase, EC:3.2.1.3). The core subdominant *Fur. rossiae* did not possess starch-hydrolyzing enzymes but showed the capability to ferment dextrin/isomaltose and maltose through oligo-1,6-glucosidase (EC:3.2.1.10) and alpha-glucosidase (EC:3.2.1.20). β-D-Glucosidase (EC:3.2.1.21) gene transcripts were unique to *Fur. rossiae*. Arabinoxylan metabolism leading to D-xylose and arabinose was exclusively found in *Fur. rossiae*.

Maltose phosphorylase (EC:2.4.1.8), glucokinase (EC:2.7.1.2) and β-phosphoglucomutase (EC:5.4.2.6) were encoded by all core dominant and subdominant lactic acid bacteria. Concomitantly, *Sac. cerevisiae* contributed to the metabolism of dextrin via maltose phosphorylase and uniquely showed the capability to catabolize maltose by amylomaltase (E. C 2.4.1.25). *Lac. plantarum* and *Ped. pentosaceus* showed the capability to use alternative energy sources such as trehalose and cellobiose via trehalose phosphorylase (EC:2.4.1.64) (only *Lac. plantarum)*, 6-phospho-beta-glucosidase (EC:3.2.1.86) and trehalose-6-phosphate hydrolase (EC:3.2.1.93). The gene encoding β-fructofuranosidase (EC:3.2.1.26), which participates in fructan metabolism, was detected in all the core dominant species and *Sta. epidermidis*. Fructokinase (EC:2.7.1.4), which metabolizes fructose, was shared by all lactic acid bacteria. Within the fructose pathway, supplementary activity was shown by yeasts through hexokinase (EC:2.7.1.1). As observed downstream of the UDP-glucose intermediate, the contribution of *Sac. cerevisiae* was often redundant with that of *Pic. kudriavzevii*. The synergistic activity of endo-xylanase (EC:3.2.1.8), β-d-xylosidase (EC:3.2.1.37), β-glucosidase (EC:3.2.1.21) and α-arabinofuranosidase (EC:3.2.1.55) was mainly expressed by *Fur. rossiae* (Fig. 4b). Regarding the fermentation of hexoses and pentoses and the alternative fate of pyruvate (Fig. 4b), the genes encoding members of the pyruvate dehydrogenase complex [pyruvate dehydrogenase (EC:1.2.4.1), dihydrolipoamide dehydrogenase (EC:1.8.1.4), pyruvate dihydrolipoamide acetyltransferase (EC:2.3.1.12)] were detected in core dominant and subdominant strains as well as in *Sta. epidermidis*. This latter also provided a supplementary contribution with D-lactate dehydrogenase (EC:1.1.1.28). L-lactate dehydrogenase (cytochrome) (EC:1.1.2.3), D-lactate dehydrogenase (cytochrome) (EC:1.1.2.4) and (R)-2-hydroxyglutarate-pyruvate transhydrogenase (EC:1.1.99.40) activities were detected only in yeasts. Yeasts and *Sta. epidermidis* encompassed the encoding gene for acetyl-CoA synthetase (EC:6.2.1.1), an enzyme using acetate to synthesize acetyl-CoA. Another alternative pathway leading to the synthesis of acetate was detected. This included pyruvate decarboxylase (EC:4.1.1.1) and aldehyde dehydrogenase NAD( +)/NADP( +) (EC:1.2.1.3/1.2.1.5). *Sac. cerevisiae* encodes a mannitol 2-dehydrogenase (EC:1.1.1.67) that reduces D-fructose to D-mannitol. An alternative mechanism for NAD^+^ regeneration was found in *Lac. plantarum,* involving mannitol-1-phosphate 5-dehydrogenase (EC:1.1.1.17), which reduces D-fructose 6-phosphate to D-mannitol 1-phosphate.

**Fig. 5:**
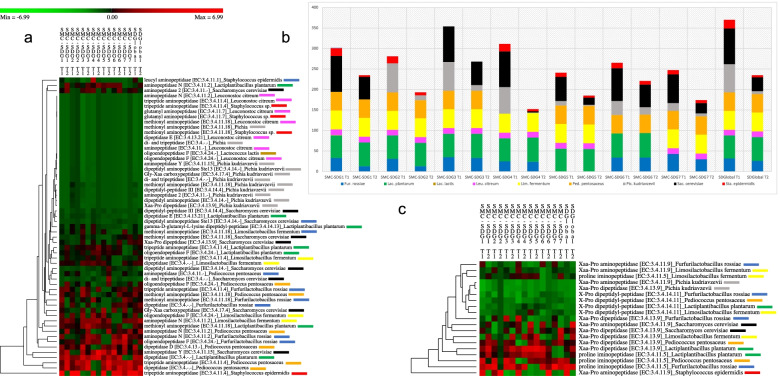
Peptidase gene transcripts in SMCs. SMC peptidase KEGG gene transcripts at exponential (T1) and stationary (T2) phases: a) permutation matrix of endo-, amino- and dipeptidases. Normalized transcript (TPM) abundances ranged from –6.99 (green) to + 6.99 (red). b) Stacked bar-plot histograms reporting the per-species gene transcripts encoding peptidases within each synthetic microbial community; and c) permutation matrix of proline-specific peptidases. Normalized transcript (TPM) abundances ranged from –4.12 (green) to + 4.12 (red)

Transcriptomic evidence of unique species-specific contributions also emerged for nitrogen metabolism (Fig. 5). The contribution of *Sta. epidermidis* is mainly related to tripeptide aminopeptidase (EC:3.4.11.4) and Xaa-pro aminopeptidase (EC:3.4.11.9). Despite the few genes transcribed, this satellite species showed higher expression levels than the other SDG species (Fig. 5a and c), whereas its global contribution was lowest (Fig. 5b). The SDG analysis showed that most nitrogen metabolism functions were shared among bacteria, while this redundancy might be extended to yeasts for only some pathways. Analysis of the BCAA metabolism pathway revealed that various enzymes were actively transcribed only in *Sac. cerevisiae* and *Pic. kudriavzevii*, which, together with *Sta. epidermidis*, contributed to several indispensable pathways. This is the case for glutamate dehydrogenase (EC:1.4.1.2) and aldehyde dehydrogenase (NAD +) (EC:1.2.1.3) (Fig. 4c). Gdh was encoded only by *Lac. plantarum, Sta. epidermidis* and yeasts. Aspartate ammonia-lyase (EC:4.3.1.1), the enzyme that converts aspartate into fumarate, was uniquely transcribed by *Lac. plantarum* (Fig. 4d). The 2 routes for arginine degradation, the arginine-urease and ADI pathways, were detected. The gene encoding arginase is present in *Lim. fermentum*, *Sac. cerevisiae* and *Pic. kudriavzevii*. Clustered genes for the ADI pathway were found in *Lim. fermentum*, *Fur. rossiae* and *Ped. pentosaceus*.

### The de novo synthetic community shows transcriptome redundancies

**Fig. 6:**
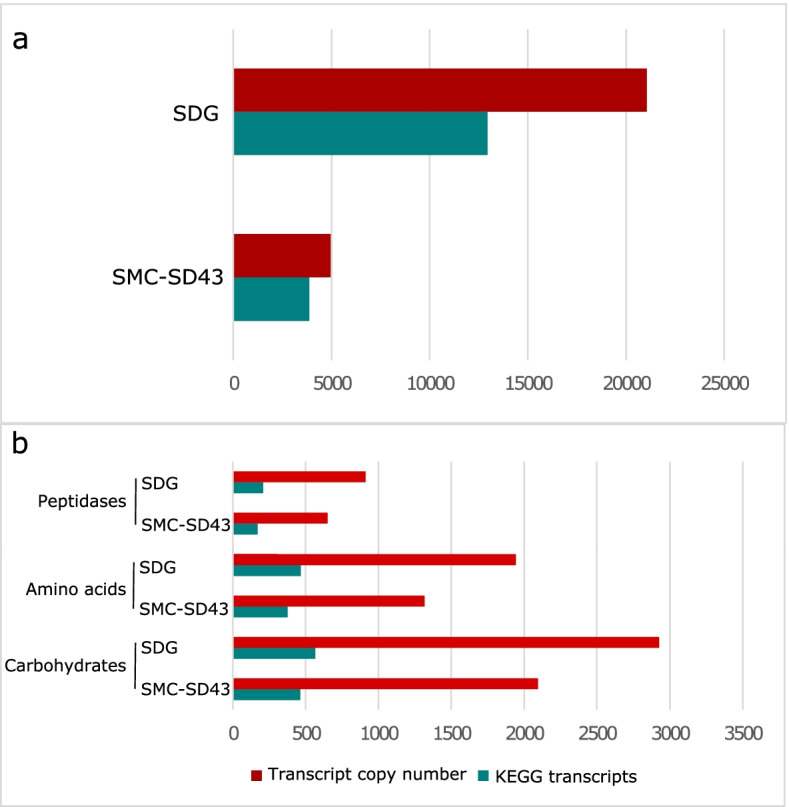
Comparison of KEGG transcripts and copy numbers between SDG and SMC-SD43. The annotated functions from the metatranscriptomes were used to assess the robustness of SDG- and SD43-reconstructed synthetic microbial communities. The occurrence (presence/absence) of transcribed functions in terms of KEGG enzymes and relative copy number have been evaluated in terms of the whole metabolic pattern (panel a) and four selected submetabolisms (panel b), inclusive of carbohydrates, amino acids, stress, and peptidases. The transcript and copy numbers are shown in purple and dark green, respectively

Together with de novo SDG, we reconstructed another synthetic microbial community (SMC-SD43) to compare transcriptomics data in exponential and stationary growth conditions. SMC-SD43 comprised all bacterial and yeast species that were detected by culturomics in the spontaneous sourdough SD43. Both SDG and SMC-SD43 were cultivated in WFH, and their levels of transcripts assigned to KEGG functions (including copies of each transcript) were assessed (Fig. 6 a and b). The data focused on the robustness of the SDG with respect to SMC-SD43. The number of total KEGG transcripts in SDG was 4 times higher than that in SMC-SD43. This overall trend was also confirmed for each of the main reconstructed pathways. The highest number of gene transcripts in SDG belonged to carbohydrate metabolism, which was represented by more than 2500 gene copies.

### The de novo synthetic community shows steady state under in situ conditions

**Fig. 7:**
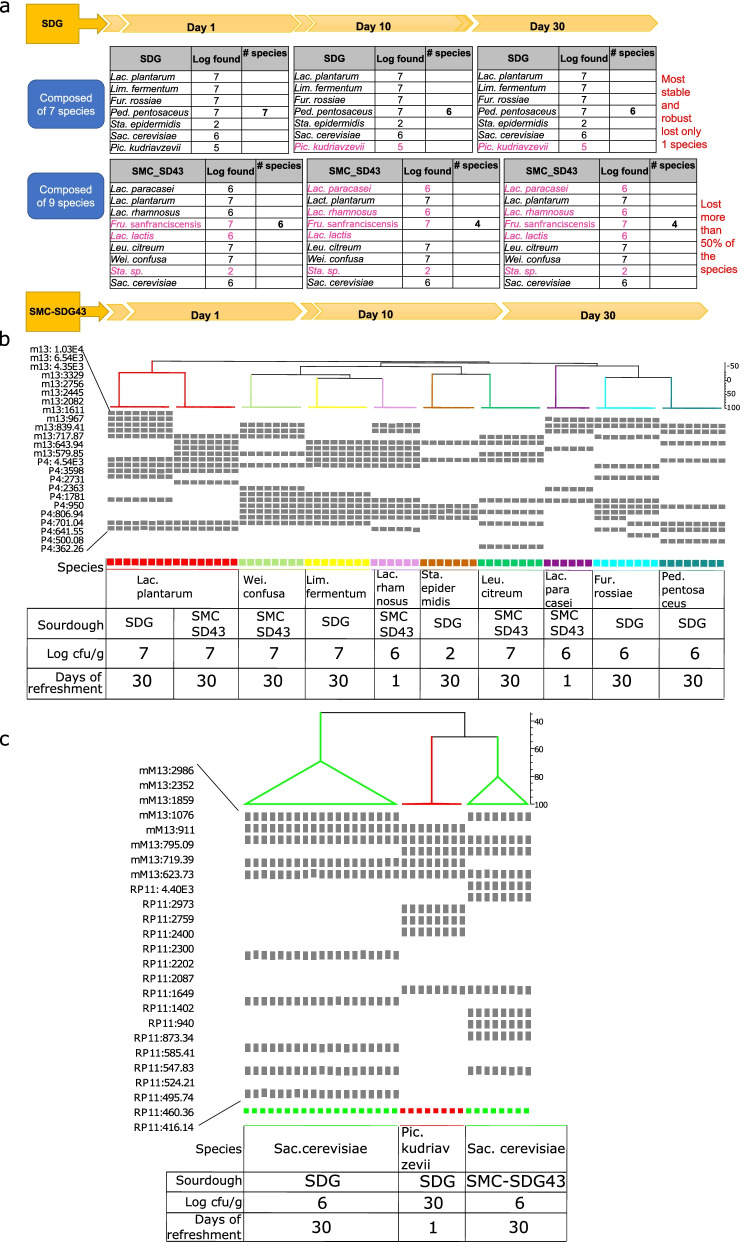
Biotypes of lactic acid bacteria and yeasts comprising SDG and SMC-SD43 during daily back slopping, as identified by RAPD-PCR. Cumulative representation of the composition and evolution of SDG and SMC-SD43 during the persistence experiment (a); pink font indicates species that were lost during back slopping. DNA fingerprinting profiles of lactic acid bacteria isolates obtained by using P4 and M13 arbitrary primers (b). DNA fingerprinting profiles of yeasts obtained by using mM13 and RP11 arbitrary primers (c). The size of the bands (base pair number) compared to the reference lane has been reported for each primer pair. On the horizontal axis, the cluster analysis of the composite profiles using the UPGMA method (BioNumerics) is given. Information about the last day the biotype was identified and in which penultimate dilution (expressed as log cfu/g) have been shown as tables. The first profile of each different strain is the reference profile of the strains used

**Fig. 8:**
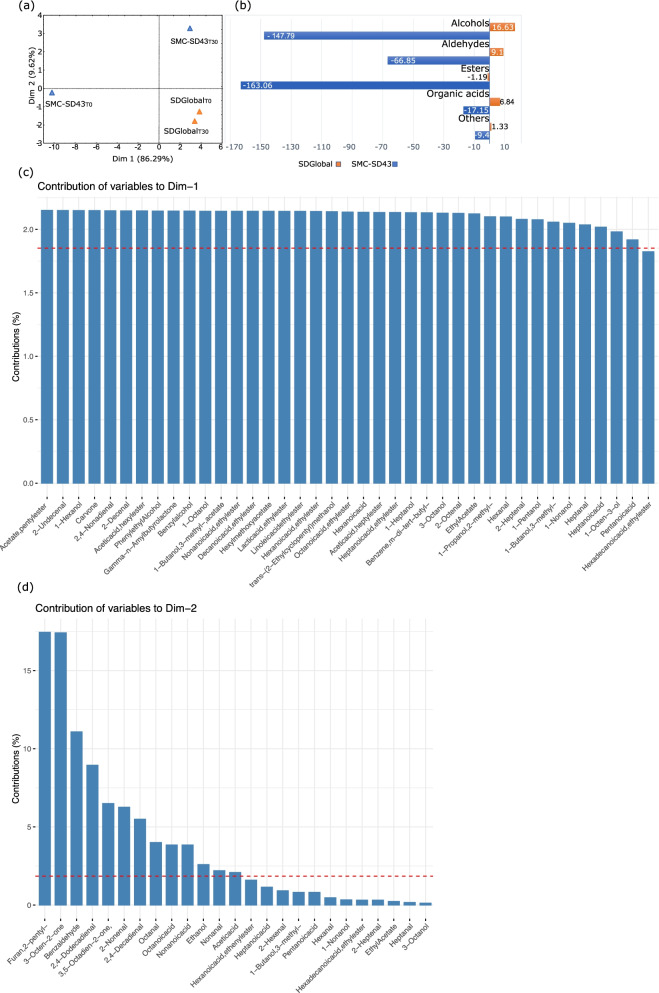
Principal component analysis of VOC and metabolic delta of chemical classes in SDG and SMC-SD43. The volatile profile normalized data matrix for SDGlobal and SMC-SD43 communities at one (T0) and thirty (T30) days of propagation was used to calculate the principal components (Dim—1 and Dim —2) reported in the PCA graph (panel a). The contributions of variables to both dimensions are reported in panels c and d. Only statistically significant VOC differences ( g/g) were used to calculate the metabolic delta of the two timepoints (T30 minus T0). The resulting metabolic delta of VOC abundances was grouped according to chemical class, e.g., alcohols, aldehydes, esters, organic acids and others (panel b)

Next, we assessed SDG and SMC-SD43 during 30 days of daily back slopping under in situ sourdough conditions. Both stabilized at similar pH values (4.3 ± 0.03 and 4.2 ± 0.02) after 5 days of propagation. The kinetics of acidification remained almost constant throughout 30 days. The cell densities of presumptive lactic acid bacteria were also steady, reaching similar values (ca. 8.51 to 8.65 log cfu/g). Presumptive staphylococci and micrococci (ca. 3.0 log cfu/g) and enterobacteria (ca. 3.9 to 4.9 log cfu/g) did not vary significantly. Yeasts slightly differed, at ca. 6.82 to 6.86 ± 0.02 log cfu/g for SMC-SD43 and 7.3 to 7.70 ± 0.04 log cfu/g for SDG. Biotype profiling (Fig. 7b and c) showed the sudden disappearance of *Fru. sanfranciscensis*, *Lac. lactis* and *Staphylococcus* sp. strains from SMC-SD43. After 10 days of propagation, other biotype members, such as *Lac. paracasei* and *Lac. rhamnosus,* were no longer detectable. At 30 days, SMC-SD43 harbored only 4 of the initial strains. In contrast, all strains inoculated in SDG persisted throughout back sloping (Fig. 7a). The only exception was *Pic. kudriavzevii*, which was no longer detectable after 10 days of propagation. The GC–MS data showed a profile of fifty-four VOCs, which reflected the stability of the sourdough metacommunities. Indeed, the levels of VOCs varied substantially during the propagation of SMC-SD43, while they remained almost constant for SDG. The statistical comparison (Welch test with BH correction) between sampling revealed variations of 45 VOCs in SMC-SD43 and only 3 VOCs in SDG (Supplementary Table S11, Additional File 13). Based on the linear distance of the principal component analysis plot, the two sampling times of SDG almost overlapped in the same quadrant (third), while those from SMC-SD43 were plotted in the second and fourth quadrants (Fig. 8).

## Discussion

Deterministic drivers render the sourdough microbiome/fermentome highly variable, dynamic and often unpredictable in terms of evolution, stability and performance [43]. As further evidence of this, the representative spontaneous sourdoughs used in this study had a heterogeneous richness in culturable bacterial and yeast species that could not be appreciated simply by evaluating the cell densities of dominant members and the ratio between lactic acid bacteria and yeasts [5].

As we discussed in an earlier systematic review [5], lactic acid bacteria usually act as dominant players in sourdoughs, but we have also been able to detect several subdominant species, which together accounted for the majority of genes/transcripts. Deterministic drivers during sourdough back slopping promote the dominance of competitive species with ecological fitness over a pool of less abundant members, the role of which must not be neglected [44]. Overall, we confirmed the constant dominance of *Sac. cerevisiae* among the yeasts [45]. Acting as satellite members, other bacterial genera (e.g., staphylococci and few enterobacteria) completed the sourdough metacommunity. Repeated back slopping provides the opportunity for even unlikely contaminants to become significant members of the sourdough microbiome [46].

### Deciphering the functional redundancy of sourdough genomes and the main players actively engaged in the main metabolic pathways

Sourdoughs shared a very high number of genes in common, although numerous unique genes were also assigned to unique sourdoughs. Biochemical pathways relevant to the main properties of sourdoughs, such as carbohydrate, pyruvate, energy and nitrogen metabolism, accounted for the highest number of genes. Based on metatranscriptomics, we might assert that higher microbial richness is accompanied by higher sourdough gene redundancy. The contribution of taxonomically close members was redundant in terms of functions [47]. This evidence confirmed the findings from other microbial ecosystems, such as those of the human gut [48], animals [49], foods [50] and plants [51]. We succeeded in assigning almost all functions to reconstructed pathways, which were fundamental for the resilience and performance of the sourdough. At the same time, we underlined the individual contributions of many players within the metacommunity that are actively part of dominant, subdominant and satellite groups. As already reported when describing the interactions between *Lac. plantarum* and *Sac. cerevisiae *[52], we demonstrated, in a more complex metacommunity, how single enzyme activities might rely on unique or multiple contributions from detectable species. All *Weissella* spp. and *Lactobacillus* spp. (former taxonomic nomenclature) detectable at the metagenomics level were also transcriptionally active in sourdoughs, whereas *Lactococcus* and *Enterococcus* were not. Although frequently harbored by spontaneous sourdoughs, these last two mentioned genera might have been displaced by lactobacilli during back slopping [46]. Some other culturable bacteria (e.g., Enterobacteriaceae) also did not exhibit transcripts, probably because of acid inhibition and/or their inability to transcribe at low cell densities. *Staphylococcus* spp. are commonly isolated from cereal flours, but they do not show sufficient competitiveness for maintenance during sourdough back slopping [53]. Nevertheless, when *Sta. epidermidis* was inoculated at higher cell densities and grown in the WFH model medium, it expressed gene transcripts, even within a heterogeneous metacommunity. To complete the critical analysis of this finding, we must acknowledge that by capturing single snapshots at specific timepoints, metatranscriptomics may suffer from specific biases relevant to transcript detection and quantification. Overall, the dominant and subdominant taxa were distinguished by the difference in numbers of genes and transcripts. *Fru. sanfranciscensis*, *Lac. plantarum, Lim. fermentum*, *Ped. pentosaceus*, *Leu. citreum* and *Wei. confusa* actively contributed to almost all reactions of all reconstructed pathways. This set of species was shared by several spontaneous sourdoughs, in particular those with the highest genetic potential and numbers of transcripts. Key enzymes such as fructose-1,6-bisphosphate aldolase (EMP pathway) and phosphoketolase (phosphogluconate pathway) are broadly distributed within the sourdough metatranscriptomes [54, 55]. Phosphoketolase was invariably present, which also indicated a role for anabolic reactions. NADH-dependent mannitol dehydrogenase activity contributing to additional cofactor regeneration was found only in *Sac. cerevisiae*. Since this enzyme is also pivotal for lactic acid bacteria competitiveness, the lack of the encoding gene might be ascribed to its high sequence similarity with alcohol- and L-threonine dehydrogenases [56]. In addition, all sourdoughs harbored the gene encoding mannitol-1-phosphate 5-dehydrogenase, which was exclusively ascribed to *Lac. plantarum *[56]. Specific reactions leading to the synthesis or release of amylose, 1–3 β-glucans and trehalose were exclusively contributed by *Sac. cerevisiae* and *P. kudriavzevii.* As confirmed by constructing the synthetic microbial community, *Sac. cerevisiae* was also unique in contributing to starch breakdown through amyloglucosidase activity (EC: 3.2.1.3). Another parallel pathway leading to the synthesis of D-glucose from β-D-glucoside revealed the unique contribution of *Fur. rossiae*.

### De novo design of synthetic microbial communities and their players contributing to metabolic resilience

Based on the taxonomic classification, functional annotation and pathway inspection, we established the groundwork for selecting those species most suitable to reconstruction of a synthetic resilient and high-performing sourdough metacommunity. According to our hypotheses, subdominant genes synergistically cooperate with dominant players to express key genes, and satellite members are equipped with unique genes that are also important for the stability of the metacommunity. In summary, gene complementarity and redundancy are the main prerequisites to guarantee sourdough efficiency. The engineering of a multispecies consortium relies on a simplified and controlled environment [57], and therefore, WFH [35] was chosen as the model system. To unravel the contribution of each player to the de novo SDGs, the metatranscriptomics assessment was repeated. All the pathways reconstructed from spontaneous sourdoughs were confirmed, and the roles of individual players were deepened. Growth phases and depletion of single members from SDG did not influence the number of transcripts of *Lac. plantarum*, *Lim. fermentum* and *Ped. pentosaceus*, which contributed redundant functions, particularly for carbohydrate pathways. These species constitutively expressed enzymes for maltose utilization (e.g., maltose phosphorylase, β-phosphoglucomutase and glucokinase), which indicated an adaptation to the sourdough ecosystem, as maltose is the most abundant fermentable carbohydrate in cereal flours, but also a physiology and ecology that matched their phylogenetic placement [58]. The comparison of exponential and stationary phases of growth revealed an adaptation in terms of species contribution. Specifically, when entering the stationary phase of growth, the number of transcripts from *Sac. cerevisiae* and *Pic. kudriavzevii* decreased; this behavior might be related to a quiescence state because of the lack of carbon sources [59]. Moreover, slightly influenced by the phase of growth, the number of transcripts of *Fur. rossiae* increased when the dominant players were depleted from SDG. *Fur. rossiae* was the only species contributing to some pathways (e.g., arabinoxylans) and enzyme activities (e.g., β-D-glucosidase), which are fundamental for the rheologic, sensory and nutritional attributes of sourdough [60, 61]. Even though staphylococci are not competitive enough to grow under sourdough conditions [62], we highlighted some fundamental contributions from these bacteria, mainly to nitrogen metabolism. Staphylococci have already been used as alternatives to lactobacilli [4] to leverage their nitrogen metabolism (e.g., converting arginine into ornithine) [63] and, in general, for the uptake and release of free amino acids.

### The de novo synthetic community shows steady state under in situ conditions

Compared to another reconstructed synthetic microbial community (SMC-SD43), which mimicked the composition of a spontaneous sourdough and had a higher number of bacterial and yeast species (9 vs*.* 7), the SDG more reliably maintained all the functions needed to resiliently express carbohydrate and nitrogen metabolism. The robustness of the SDG was attributable to its higher gene expression in terms of total KEGG pathways and copy numbers. The last proof to confirm our hypotheses was based on the in situ resilience and performance, which we monitored under perturbations that occurred during the daily sourdough back slopping. As expected, the two synthetic microbial communities did not show variation in terms of acidification or numbers of presumptive lactic acid bacteria and yeasts. It has been widely demonstrated that contaminant species may replace the initial ones and that these qualitative changes within the metacommunity are not observable using simple determinations such as pH and plating [64]. Here, much more finely tuned biotype profiling revealed how the metacommunity of SDG remained stable during 30 days of propagation, while that of SMC-SD43 lost most of its species members. These changes might affect sourdough performance in terms of flavor, where VOC synthesis may be a suitable indicator. In fact, the VOC profiles of SDG overlapped during 30 days of propagation, while those of SMC-SD43 drastically changed.

## Conclusions

In our complex workflow, we envisioned the sourdough as a specialized social structure from which members colonize the new flour environment in a complementary manner that is orchestrated strategically to cover all accompanying metabolic pathways. Our study demonstrates how, by starting from spontaneous sourdoughs and reconstructing the synthetic community, it was possible to unravel the metabolic contributions of individual players. For resilience and good performance, the sourdough metacommunity needed to include dominant, subdominant and satellite players, which together ensured gene and transcript redundancy. Overall, our study changes the paradigm and introduced theoretical foundations for directing food fermentations.

### Data availability

The BioProject, including metagenomics and metatranscriptomics samples, is under submission.

## Supplementary Information

**Additional file 1: Supplementary Table S1.** Origin, ingredients and technology parameters of sourdoughs. 

**Additional file 2: Supplementary Table S2.** Assembly statistics relative to the eight spontaneous sourdough assembled metagenomes.

**Additional file 3: Supplementary Table S3.** Total number of genera and species of bacteria detected in the eight spontaneous sourdoughs by shotgun metagenomics.

**Additional file 4: Supplementary Table S4.** Total number of genera and species of yeasts detected in the eight spontaneous sourdoughs by shotgun metagenome analysis. 

**Additional file 5: Supplementary Figure S1.** Core and dispensable species found in the eight spontaneous sourdoughs by culturing, and shotgun meta-genomics and -transcriptomics. Pseudo-heatmap displaying the comparison between the core (species shared by the eight sourdoughs) and dispensable (species with relative abundance >0.1% variously detected within one or more sourdoughs) transcriptionally active microbiome species under sourdough conditions, and cultivable microbiota. Colour bar on the right describes species prevalence. Among omics C stands for culturomics, SM and MT stand for shotgun metagenomics and metatranscriptomics, respectively.

**Additional file 6: Supplementary Table S5.** Total number of mapped reads in the eight spontaneous sourdoughs by metatranscriptomics analysis.

**Additional file 7: Supplementary Table S6.** Metagenomic ORFs predicted by using Aragorn, Prodigal and Barrnap and relative ORF annotations based on the KEGG, COG and Pfam databases for each sourdough (SD). 

**Additional file 8: Supplementary Table S7.** Shared and unique KEGG genes from metagenomes in the eight spontaneous sourdoughs.

**Additional file 9: Supplementary Table S8.** List of KEGG ID, gene name, function, transcript level (transcripts per million = TPM), copy number, total bases and coverage of each gene found in sourdough metagenomes.

**Additional file 10: Supplementary Figure S2.** Gene and transcript class content (core and dispensable dominants and subdominants) for aminotransferase a) deaminase/lyase b), carbohydrate c) and pyruvate d) microbial pathways in the 8 spontaneous sourdoughs.

**Additional file 11: Supplementary Table S9.** Core and accessory genes encoding the enzymatic portfolio related to protein/peptide degradation in the eight spontaneous sourdoughs as estimated by metagenomic analyses. 

**Additional file 12: Supplementary Table S10.** Distribution of genes encoding the catabolism of branched chain amino acids (BCAAs), aromatic amino acids (ArAAs) and free amino acids. The expressed genes, evaluated through metatranscriptomic analyses, are marked by asterisks. 

**Additional file 13: Supplementary Table S11.** Statistically significant VOCs in SDGlobal and SMC-SD43. VOC standardized abundance whole matrices were used to compute a Welch corrected test (Benjamini–Hochberg). Mean rel. freq. = mean relative frequency; std.dev. = standard deviation; CI = confidence interval.

## Data Availability

The original contributions presented in the study are included in the article/Supplementary Material. Further inquiries can be directed to the corresponding author.

## References

[1] Friedman J, Higgins LM, Gore J (2017). Community structure follows simple assembly rules in microbial microcosms. Nat Ecol Evol.

[2] novel avenues for bio-based processes (2021). Diender, M., Parera Olm, I. & Sousa, D. Z. Synthetic co-cultures. Curr Opin Biotechnol.

[3] Antoniewicz MR (2020). A guide to deciphering microbial interactions and metabolic fluxes in microbiome communities. Curr Opin Biotechnol.

[4] Comasio A, Verce M, Van Kerrebroeck S, De Vuyst L (2020). Diverse Microbial Composition of Sourdoughs From Different Origins. Front Microbiol.

[5] Arora K (2021). Thirty years of knowledge on sourdough fermentation: A systematic review. Trends Food Sci Technol.

[6] Minervini F, Dinardo FR, Celano G, De Angelis M, Gobbetti M (2018). Lactic Acid Bacterium Population Dynamics in Artisan Sourdoughs Over One Year of Daily Propagations Is Mainly Driven by Flour Microbiota and Nutrients. Front Microbiol.

[7] Grosskopf T, Soyer OS (2014). Synthetic microbial communities. Curr Opin Microbiol.

[8] De Angelis M (2006). Selection of potential probiotic lactobacilli from pig feces to be used as additives in pelleted feeding. Res Microbiol.

[9] Kurtzman CP, Robnett CJ (1998). Identification and phylogeny of ascomycetous yeasts from analysis of nuclear large subunit (26S) ribosomal DNA partial sequences. Antonie Van Leeuwenhoek.

[10] Lugli GA (2019). Compositional assessment of bacterial communities in probiotic supplements by means of metagenomic techniques. Int J Food Microbiol.

[11] Wagner Mackenzie B, Waite DW, Taylor MW (2015). Evaluating variation in human gut microbiota profiles due to DNA extraction method and inter-subject differences. Front Microbiol.

[12] Fuxman Bass, J. I., Reece-Hoyes, J. S. & Walhout, A. J. M. Zymolyase-Treatment and Polymerase Chain Reaction Amplification from Genomic and Plasmid Templates from Yeast. *Cold Spring Harb Protoc* 2016, (2016). 10.1101/pdb.prot088971 PMC5458600 27934686

[13] Tamames J, Puente-Sánchez F (2018). SqueezeMeta, A Highly Portable, Fully Automatic Metagenomic Analysis Pipeline. Front Microbiol.

[14] Bolger AM, Lohse M, Usadel B (2014). Trimmomatic: a flexible trimmer for Illumina sequence data. Bioinformatics.

[15] Li D, Liu C-M, Luo R, Sadakane K, Lam T-W (2015). MEGAHIT: an ultra-fast single-node solution for large and complex metagenomics assembly via succinct de Bruijn graph. Bioinformatics.

[16] Muggli MD (2017). Succinct colored de Bruijn graphs. Bioinformatics.

[17] Schmieder R, Edwards R (2011). Quality control and preprocessing of metagenomic datasets. Bioinformatics.

[18] Seemann T (2014). Prokka: rapid prokaryotic genome annotation. Bioinformatics.

[19] Wang Q, Garrity GM, Tiedje JM, Cole JR (2007). Naive Bayesian classifier for rapid assignment of rRNA sequences into the new bacterial taxonomy. Appl Environ Microbiol.

[20] Laslett D, Canback B (2004). ARAGORN, a program to detect tRNA genes and tmRNA genes in nucleotide sequences. Nucleic Acids Res.

[21] Hyatt D (2010). Prodigal: prokaryotic gene recognition and translation initiation site identification. BMC Bioinformatics.

[22] Clark K, Karsch-Mizrachi I, Lipman DJ, Ostell J, Sayers EW (2016). GenBank. Nucleic Acids Res.

[23] Huerta-Cepas, J. *et al.* eggNOG 4.5: a hierarchical orthology framework with improved functional annotations for eukaryotic, prokaryotic and viral sequences. Nucleic Acids Res 44, D286–293 (2016). 10.1093/nar/gkv1248 PMC4702882 26582926

[24] Kanehisa M, Goto S (2000). KEGG: kyoto encyclopedia of genes and genomes. Nucleic Acids Res.

[25] Buchfink B, Xie C, Huson DH (2015). Fast and sensitive protein alignment using DIAMOND. Nat Methods.

[26] A new generation of homology search tools based on probabilistic inference - PubMed. https://pubmed.ncbi.nlm.nih.gov/20180275/. 20180275

[27] Finn RD (2016). The Pfam protein families database: towards a more sustainable future. Nucleic Acids Res.

[28] Langmead B, Salzberg SL (2012). Fast gapped-read alignment with Bowtie 2. Nat Methods.

[29] Wu, Y.-W., Simmons, B. A. & Singer, S. W. MaxBin 2.0: an automated binning algorithm to recover genomes from multiple metagenomic datasets. Bioinformatics 32, 605–607 (2016). 10.1093/bioinformatics/btv638 26515820

[30] Kang DD (2019). MetaBAT 2: an adaptive binning algorithm for robust and efficient genome reconstruction from metagenome assemblies. PeerJ.

[31] Sieber CMK (2018). Recovery of genomes from metagenomes via a dereplication, aggregation and scoring strategy. Nat Microbiol.

[32] Parks DH, Imelfort M, Skennerton CT, Hugenholtz P, Tyson GW (2015). CheckM: assessing the quality of microbial genomes recovered from isolates, single cells, and metagenomes. Genome Res.

[33] Ye Y, Doak TG (2009). A parsimony approach to biological pathway reconstruction/inference for genomes and metagenomes. PLoS Comput Biol.

[34] Zapparoli G, Torriani S, Pesente P, Dellaglio F (1998). Design and evaluation of malolactic enzyme gene targeted primers for rapid identification and detection of Oenococcus oeni in wine. Lett Appl Microbiol.

[35] Di Cagno R (2007). Cell-cell communication in sourdough lactic acid bacteria: a proteomic study in Lactobacillus sanfranciscensis CB1. Proteomics.

[36] Zwietering, M. H., Jongenburger, I., Rombouts, F. M. & van ’t Riet, K. Modeling of the bacterial growth curve. Appl Environ Microbiol 56, 1875–1881 (1990). 10.1128/aem.56.6.1875-1881.1990 PMC184525 16348228

[37] Parks DH, Tyson GW, Hugenholtz P, Beiko RG (2014). STAMP: statistical analysis of taxonomic and functional profiles. Bioinformatics.

[38] Liu G (2020). Headspace solid-phase microextraction of semi-volatile ultraviolet filters based on a superhydrophobic metal-organic framework stable in high-temperature steam. Talanta.

[39] Perri G (2021). Bioprocessing of Barley and Lentil Grains to Obtain In Situ Synthesis of Exopolysaccharides and Composite Wheat Bread with Improved Texture and Health Properties. Foods.

[40] Ercolini D (2013). Microbial Ecology Dynamics during Rye and Wheat Sourdough Preparation. Appl Environ Microbiol.

[41] Michel E (2016). Characterization of relative abundance of lactic acid bacteria species in French organic sourdough by cultural, qPCR and MiSeq high-throughput sequencing methods. Int J Food Microbiol.

[42] Menezes, L. a. A. *et al.* Sourdough bacterial dynamics revealed by metagenomic analysis in Brazil. Food Microbiol 85, 103302 (2020). 10.1016/j.fm.2019.103302 31500708

[43] Oshiro M, Tanaka M, Momoda R, Zendo T, Nakayama J (2021). Mechanistic Insight into Yeast Bloom in a Lactic Acid Bacteria Relaying-Community in the Start of Sourdough Microbiota Evolution. Microbiol Spectr.

[44] Gänzle M, Ripari V (2016). Composition and function of sourdough microbiota: From ecological theory to bread quality. Int J Food Microbiol.

[45] Lau SW, Chong AQ, Chin NL, Talib RA, Basha RK (2021). Sourdough Microbiome Comparison and Benefits Microorganisms.

[46] Gänzle MG, Zheng J (2019). Lifestyles of sourdough lactobacilli - Do they matter for microbial ecology and bread quality?. Int J Food Microbiol.

[47] Louca S (2018). Function and functional redundancy in microbial systems. Nat Ecol Evol.

[48] Moya A, Ferrer M (2016). Functional Redundancy-Induced Stability of Gut Microbiota Subjected to Disturbance. Trends Microbiol.

[49] Estrada-Peña A, Cabezas-Cruz A, Obregón D (2020). Behind Taxonomic Variability: The Functional Redundancy in the Tick Microbiome. Microorganisms.

[50] Kothe CI (2021). Unraveling the world of halophilic and halotolerant bacteria in cheese by combining cultural, genomic and metagenomic approaches. Int J Food Microbiol.

[51] Harbort CJ (2020). Root-Secreted Coumarins and the Microbiota Interact to Improve Iron Nutrition in Arabidopsis. Cell Host Microbe.

[52] Zhang G (2021). Proteomic Analysis Explores Interactions between Lactiplantibacillus plantarum and Saccharomyces cerevisiae during Sourdough Fermentation. Microorganisms.

[53] De Vuyst L, Van Kerrebroeck S, Leroy F (2017). Microbial Ecology and Process Technology of Sourdough Fermentation. Adv Appl Microbiol.

[54] Weckx S (2010). Community dynamics of bacteria in sourdough fermentations as revealed by their metatranscriptome. Appl Environ Microbiol.

[55] De Vuyst, L., Comasio, A. & Kerrebroeck, S. V. Sourdough production: fermentation strategies, microbial ecology, and use of non-flour ingredients. *Crit Rev Food Sci Nutr* 1–33 (2021) doi:10.1080/10408398.2021.1976100. 10.1080/10408398.2021.1976100 34523363

[56] Weckx S, Van Kerrebroeck S, De Vuyst L (2019). Omics approaches to understand sourdough fermentation processes. Int J Food Microbiol.

[57] Zhang Y, Kastman EK, Guasto JS, Wolfe BE (2018). Fungal networks shape dynamics of bacterial dispersal and community assembly in cheese rind microbiomes. Nat Commun.

[58] Zheng J, Ruan L, Sun M, Gänzle M (2015). A Genomic View of Lactobacilli and Pediococci Demonstrates that Phylogeny Matches Ecology and Physiology. Appl Environ Microbiol.

[59] Martinez MJ (2004). Genomic analysis of stationary-phase and exit in Saccharomyces cerevisiae: gene expression and identification of novel essential genes. Mol Biol Cell.

[60] Pontonio E (2016). Cloning, expression and characterization of a β-D-xylosidase from Lactobacillus rossiae DSM 15814(T). Microb Cell Fact.

[61] Son S-H (2018). Probiotic lactic acid bacteria isolated from traditional Korean fermented foods based on β-glucosidase activity. Food Sci Biotechnol.

[62] De Vuyst L, Van Kerrebroeck S, Leroy F (2017). Microbial Ecology and Process Technology of Sourdough Fermentation. Adv Appl Microbiol.

[63] Sánchez Mainar, M., Weckx, S. & Leroy, F. Coagulase-negative Staphylococci favor conversion of arginine into ornithine despite a widespread genetic potential for nitric oxide synthase activity. Appl Environ Microbiol 80, 7741–7751 (2014). 10.1128/AEM.02298-14 PMC4249236 25281381

[64] Gobbetti M, Minervini F, Pontonio E, Di Cagno R, De Angelis M (2016). Drivers for the establishment and composition of the sourdough lactic acid bacteria biota. Int J Food Microbiol.

