# Supplementary material for: Metabolic framework of spontaneous and synthetic sourdough metacommunities to reveal microbial players responsible for resilience and performance
Source: Microbiome. 2022 Sep 14;10:148. doi: 10.1186/s40168-022-01301-3 (PMC9472446; doi:10.1186/s40168-022-01301-3)
Supplement: Supplementary file 2 — Additional file 1: Supplementary Table S1. Origin, ingredients and technology parameters of sourdoughs. [file 40168_2022_1301_MOESM1_ESM.docx]

**Supplementary Table S1.** Origin, ingredients and technology parameters of sourdoughs.

| **Sourdoughs** | **Country of origin** | **Sourdough name** | **Flour** | **Specific ingredients (start up or back slopping)** | **Dough Yield** | **Back slopping temperature** | **Back slopping time** | **1^st^ Back slopping % of inoculum** | **2^nd^ Back slopping % of inoculum** |
| --- | --- | --- | --- | --- | --- | --- | --- | --- | --- |
| SD1 | Italy | Pane di Altamura PDO | Durum wheat | - | 209 | 18°C | 24 h | 40 | 10 |
| SD43 | USA | San Francisco sourdough | Strong wheat | - | 150 | 25°C | 3 h | 40 | 50 |
| SD44 | France | Kayser liquid | Soft wheat | Honey and T80^*^ | 200 | 28°C | 5 h | 40 | 33 |
| SD69 | Italy | Madre | Soft wheat | - | 143 | 8°C | 24 h | 40 | 26 |
| SD88 | Spain | Saint Honoré | Soft wheat flour T80* | Beer | 200 | 26°C | 3 h | 40 | 11 |
| SD93 | Spain | Rebola MMC | Soft wheat | - | 160 | 24°C | 2 h | 40 | 38 |
| SD102 | USA | Levain James | Soft wheat | - | 200 | 12°C | 16 h | 40 | 8 |
| SD104 | USA | That's a Some Pizza | Strong wheat | - | 200 | 10°C | 12 h | 40 | 50 |

*“Type 80” refers to the amount of Ash – bits of bran and germ – intentionally left in the flour after it has been milled.
