# Supplementary material for: Metabolic framework of spontaneous and synthetic sourdough metacommunities to reveal microbial players responsible for resilience and performance
Source: Microbiome. 2022 Sep 14;10:148. doi: 10.1186/s40168-022-01301-3 (PMC9472446; doi:10.1186/s40168-022-01301-3)
Supplement: Supplementary file 3 — Additional file 2: Supplementary Table S2. Assembly statistics relative to the eight spontaneous sourdough assembled metagenomes. [file 40168_2022_1301_MOESM2_ESM.docx]

**Supplementary Table S2.** Assembly statistics relative to the eight spontaneous sourdough assembled metagenomes.

| **Summary of Assembly statistics** | **Numeric/percentage/rank** |
| --- | --- |
| Number of contigs | 6936700 |
| Total length | 2837404593 |
| Longest contig | 244981 |
| Shortest contig | 200 |
| N50 (bp) | 813 |
| N75 (bp) | 674 |
| N90 (bp) | 516 |
| N95 (bp) | 401 |
| Contigs at superkingdom (k) rank | 3972689 (57.2%), in 3 superkingdoms |
| Contigs at phylum (p) rank | 3879103 (55.9%), in 34 phyla |
| Contigs at class (c) rank | 3750100 (54.6%), in 57 classes |
| Contigs at order (p) rank | 3761006 (54.2%), in 121 orders |
| Contigs at family (f) rank | 3678455 (53.2%), in 195 families |
| Contigs at genus (g) rank | 3487904 (50.0%), in 333 genera |
| Contigs at species (s) rank | 2666755 (38.4%), in 385 species |
