# Supplementary figures and images for: Metabolic framework of spontaneous and synthetic sourdough metacommunities to reveal microbial players responsible for resilience and performance
Source: Microbiome. 2022 Sep 14;10:148. doi: 10.1186/s40168-022-01301-3 (PMC9472446; doi:10.1186/s40168-022-01301-3)

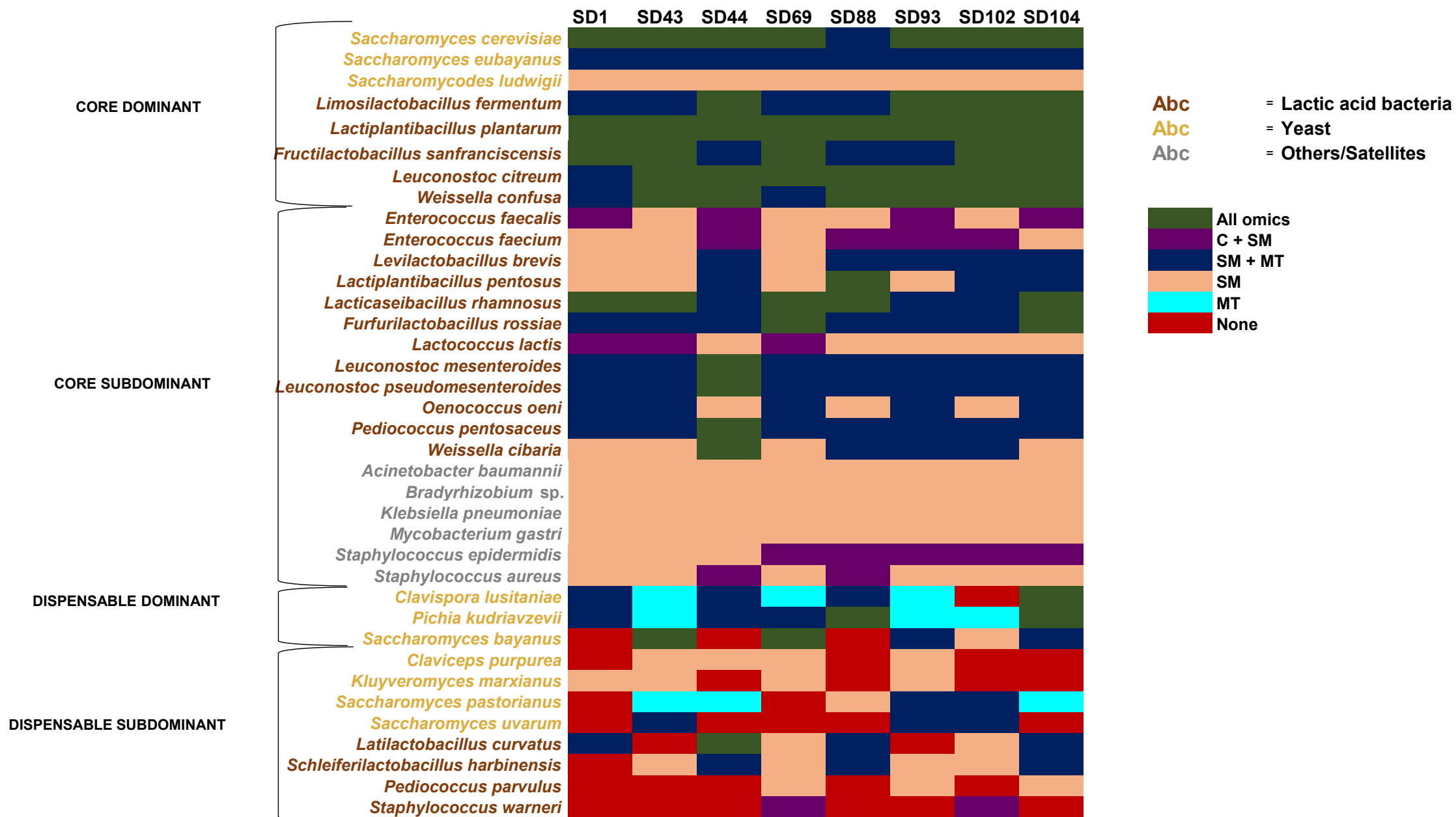

Supplement: Supplementary file 6 — Additional file 5: Supplementary Figure S1. Core and dispensable species found in the eight spontaneous sourdoughs by culturing, and shotgun meta-genomics and -transcriptomics. Pseudo-heatmap displaying the comparison between the core (species shared by the eight sourdoughs) and dispensable (species with relative abundance >0.1% variously detected within one or more sourdoughs) transcriptionally active microbiome species under sourdough conditions, and cultivable microbiota. Colour bar on the right describes species prevalence. Among omics C stands for culturomics, SM and MT stand for shotgun metagenomics and metatranscriptomics, respectively. [file 40168_2022_1301_MOESM5_ESM.pdf]

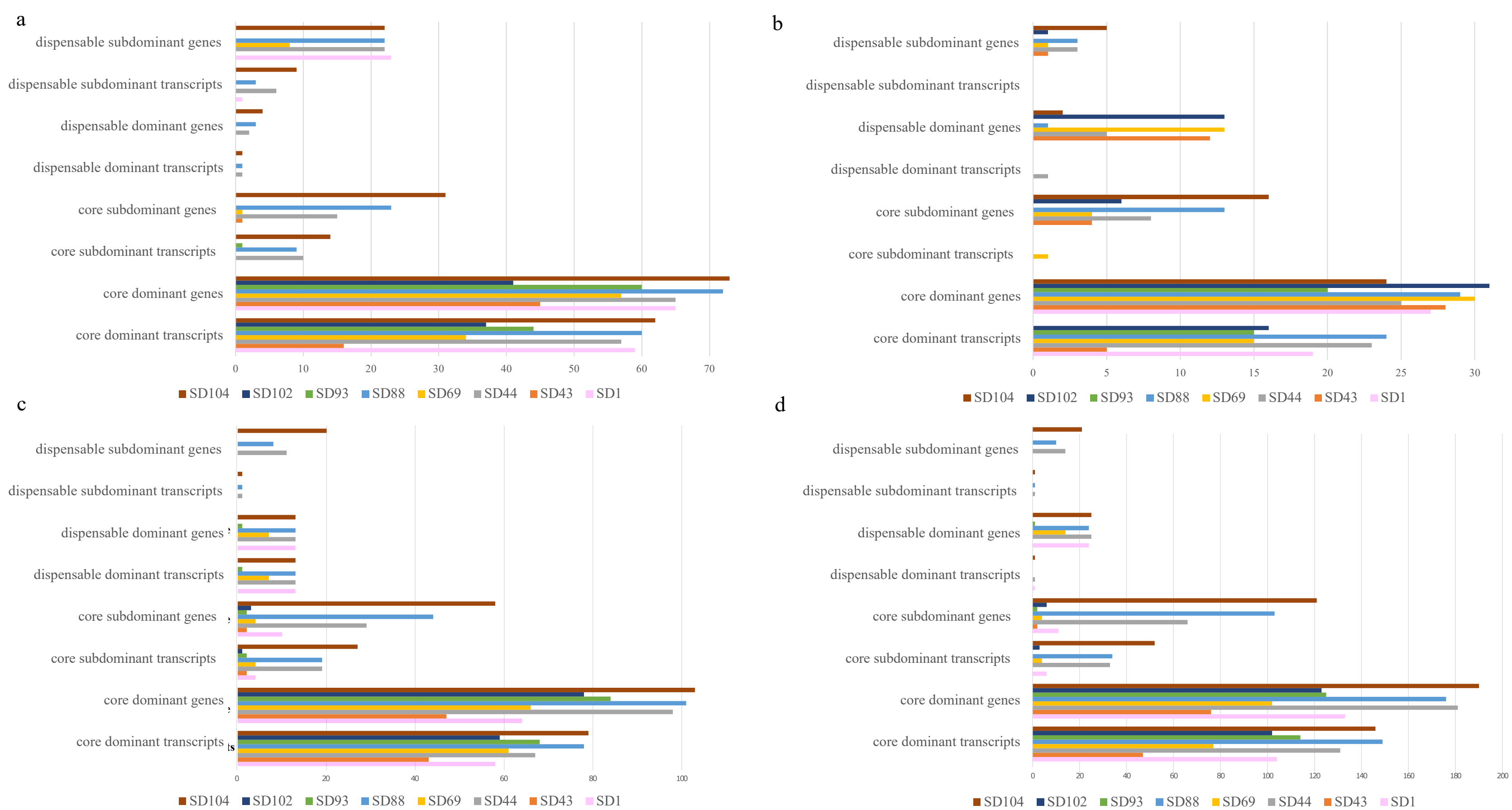

Supplement: Supplementary file 11 — Additional file 10: Supplementary Figure S2. Gene and transcript class content (core and dispensable dominants and subdominants) for aminotransferase a) deaminase/lyase b), carbohydrate c) and pyruvate d) microbial pathways in the 8 spontaneous sourdoughs. [file 40168_2022_1301_MOESM10_ESM.pdf]
