# Supplementary material for: Metabolic framework of spontaneous and synthetic sourdough metacommunities to reveal microbial players responsible for resilience and performance
Source: Microbiome. 2022 Sep 14;10:148. doi: 10.1186/s40168-022-01301-3 (PMC9472446; doi:10.1186/s40168-022-01301-3)
Supplement: Supplementary file 8 — Additional file 7: Supplementary Table S6. Metagenomic ORFs predicted by using Aragorn, Prodigal and Barrnap and relative ORF annotations based on the KEGG, COG and Pfam databases for each sourdough (SD). [file 40168_2022_1301_MOESM7_ESM.docx]

**Supplementary** **table S6.** Metagenomic ORFs predicted by using Aragorn, Prodigal and barrnap and relative ORF annotations based on KEGG, COG and Pfam databases for each one of the sourdoughs (SD).

| **ORF** | **Assembly** | **SD1** | **SD43** | **SD44** | **SD69** | **SD88** | **SD93** | **SD102** | **SD104** |
| --- | --- | --- | --- | --- | --- | --- | --- | --- | --- |
| ORFs | 5436821 | 1949761 | 779660 | 237161 | 1102090 | 421306 | 1027018 | 170771 | 404170 |
| rRNAs | 13267 | 4210 | 3554 | 2463 | 3480 | 3187 | 4462 | 3022 | 4188 |
| tRNAs | 7385 | 1446 | 1014 | 1375 | 934 | 1828 | 1145 | 1358 | 2195 |
| ORFs by Aragorn | 7385 | 1446 | 1014 | 1375 | 934 | 1828 | 1145 | 1358 | 2195 |
| ORFs by Prodigal | 5416169 | 1944105 | 775092 | 233323 | 1097676 | 416291 | 1021411 | 166391 | 397787 |
| ORFs by barrnap | 13267 | 4210 | 3554 | 2463 | 3480 | 3187 | 4462 | 3022 | 4188 |
| Orphans (no hits) | 3222020 | 1234911 | 460180 | 96049 | 670652 | 211764 | 611897 | 63117 | 167217 |
| No tax assigned | 86559 | 24907 | 15784 | 2009 | 20608 | 4602 | 19063 | 505 | 3074 |
| KEGG annotations | 550922 | 107999 | 69529 | 68447 | 70217 | 84481 | 74903 | 61292 | 111382 |
| COG annotations | 1090978 | 287558 | 149052 | 102128 | 185002 | 132227 | 183028 | 82087 | 164645 |
| Pfam annotations | 154343 | 49350 | 22909 | 28872 | 33051 | 35932 | 34466 | 17694 | 44204 |
